# Early sex-related transcriptional differences in CD8^+^ T cells responding to chronic viral infection reveal a sex bias in exhaustion

**DOI:** 10.3389/fimmu.2026.1783098

**Published:** 2026-05-12

**Authors:** Nyambura Kahia, Sean M. Robertson, Megan Rodriguez, Paul Jerard Layug, Harman Vats, Kamali Kannan, Victor Spicer, Arzu Ozturk-Aptekmann, Olivia Wilkins, Janilyn Arsenio

**Affiliations:** 1 Department of Immunology, University of Manitoba, Winnipeg, MB, Canada; 2 Biomedical Proteomics, Department of Internal Medicine Manitoba Centre for Proteomics and Systems Biology, Winnipeg, MB, Canada; 3 Department of Biological Sciences, University of Manitoba, Winnipeg, MB, Canada; 4 Department of Internal Medicine, University of Manitoba, Winnipeg, MB, Canada

**Keywords:** CD8 T cells, infection, scRNA-seq, sex differences, T cell exhaustion

## Abstract

CD8^+^ T cell diversity is essential to control infections and chronic antigen stimulation. In acute-resolving infection, effector cells mediate acute responses and memory cells provide long-lived protection against future exposures. In chronic infection and cancer, an altered state called exhaustion occurs. Exhausted CD8^+^ T cells are molecularly and functionally distinct from effector and memory cells. Differences in immune responses exist between biological sexes, however, how biological sex influences the timing and transcriptional programs of CD8^+^ T cell responses during chronic versus acute viral infection remains unknown. Here, we show that male and female CD8^+^ T cells exhibit transcriptional differences in their early responses during chronic but not acute viral infection *in vivo*. Using single-cell RNA-sequencing and immunophenotyping analyses, we show that female CD8^+^ T cells exhibit an early exhaustion-like program compared to males. These findings reveal new insights into sex-related differences in CD8^+^ T cell exhaustion development and early T cell responses that may contribute to sex differential immune responses.

## 1 Introduction

CD8^+^ T cell functional diversity is integral to controlling infections and chronic antigen stimulation. During the immune response to an acute infection, responding CD8^+^ T cells differentiate into effector and memory cells. Effector CD8^+^ T cells are cytotoxic, secrete pro-inflammatory cytokines, such as IFN-γ and TNF-α, granzymes, and perforins to rapidly clear infected cells, whereas circulating and tissue-resident memory CD8^+^ T cells respond to recurrent infections (1–6). In states of chronic antigen stimulation, such as chronic infection and cancer, CD8^+^ T cells adopt an altered functional state called exhaustion, characterized by a progressive loss in effector functions, reduced cytokine production, and high co-expression of inhibitory receptors such as PD-1, LAG3, TIM3, and CTLA-4 (7). The well-characterized *lymphocytic choriomeningitis* (LCMV) model system has been instrumental in studying CD8^+^ T cell responses in acute and chronic infections (6, 8). Acute-resolving infection with the LCMV-Armstrong strain leads to the generation of effector and memory CD8^+^ T cells, whereas chronic infection with the LCMV-clone 13 strain results in chronic antigen persistence and CD8^+^ T cell exhaustion. Exhausted CD8^+^ T cell heterogeneity, including progenitor, transitory or intermediate, and terminal differentiated states, have been defined by their altered transcriptional and epigenetic programs, and functional differences relative to effector and memory CD8^+^ T cells using the LCMV model (8–10). While the exact timing of when the specification of an exhaustion or effector/memory program of CD8^+^ T cell differentiation occurs remains to be fully understood (11), CD8^+^ T cells displayed hallmarks of exhaustion as early as after the first cell division after LCMV-clone 13 infection (12), and within hours after encountering tumor antigen (13).

Biological sex differences occur in innate and adaptive immune responses to infection, autoimmunity, malignancies (14), in the progression of chronic viral infections (15, 16), and in responses to treatment (17); however, the underlying cellular mechanisms contributing to these differences remain incompletely defined. In general, females mount stronger innate and antibody responses to infection and have a higher prevalence of inflammatory and autoimmune diseases compared to males (14). Sex differences in the immune system can be genome encoded or mediated by sex hormones (14) that can act directly or indirectly on T lymphocytes (18, 19). Sex biases in CD8^+^ T cell responses may be context dependent. In response to acute *vaccinia virus* or *Listeria* infection (20), and Coxsackievirus B3 infection (21), greater effector CD8^+^ T cell expansion was observed in females, whereas in response to stroke, effector CD8^+^ T cell activities were increased in male mice (22). In non-reproductive cancers with known higher prevalence in males, androgen receptor expression correlated with increased tumor-infiltrating exhausted CD8^+^ T cells in males (23–25), while cell-intrinsic regulatory mechanisms mediated an increase of male exhausted CD8^+^ T cells in glioblastoma (26) and colorectal cancer (27). Overall, these studies underscore a critical role of biological sex in diverse CD8^+^ T cell-mediated immune responses. However, a paucity of sex-disaggregated data analysis in prior CD8^+^ T cell differentiation studies has precluded the ability to discern how biological sex influences CD8^+^ T cell responses, their timing and molecular programs during acute versus chronic infection.

Here, we characterize previously unexplored biological sex-related differences in CD8^+^ T cell responses and their transcriptional programs during acute versus chronic LCMV infection. In the blood of both infection settings, we found that male responding CD8^+^ T cells exhibit increased expansion capacities compared to female responding CD8^+^ T cells. Greater pro-inflammatory cytokine expression was observed in female compared to males in both infection types. Using single cell RNA-sequencing (scRNA-seq), we identified sex-related transcriptional differences early in chronic, but not acute infection. Intriguingly, female responding CD8^+^ T cells exhibited an early exhaustion-like transcriptional program and a greater exhaustion phenotype after *in vivo* chronic infection compared to their male counterparts, suggesting that female CD8^+^ T cells may adopt an early program of exhaustion differentiation. These findings provide novel insight into biological sex differences on early CD8^+^ T cell exhaustion development.

## 2 Methods

### 2.1 Mice

All animal work was approved by the Animal Care Committee of the University of Manitoba, Winnipeg, Canada. All mice were bred and housed in specific pathogen-free conditions. Congenic marked CD45.1 C57BL/6 mice and CD45.2 P14 C57BL/6 mice, which have T cell receptors specific for the LCMV glycoprotein 33–41 peptide residues (GP_33-41_) in the context of H-2D^b^, were purchased from The Jackson Laboratory. CD45.1 C57BL/6 mice were crossed with CD45.2 P14 transgenic mice at the University of Manitoba to obtain CD45.1 P14 donor mice. Donor and recipient wild-type CD45.2 C57BL/6 mice were male or female, 6–8 weeks old. Donor P14 CD45.1^+^ female and male mice were progeny from the same dam and sire. Recipient CD45.2 female or male mice were housed in same-sex cages. Donor and recipient mice were age- and sex-matched. For infection experiments, no randomization or blinding was used.

### 2.2 Antibodies and flow cytometry

Antibodies (BioLegend) included: CD8α (53-6.7), CD45.1 (A20), CD62L (MEL-14), CD44 (1M7), IL-2Rα (PC61), PD-1 (RMP1-30), Ki-67 (11-F6), IFN-γ (XMG1.2), TNF (MP6-XT22), TIM-3 (B8.2C12), Ly108 (330-AJ), IL-2 (JES6-5H4), KLRG1 (2F1/KLRG1), IL-7R α (SB/199), LAG-3 (C9B7W), Annexin V-PE, and TCF-1 (S33-966) from BD Pharmingen Biosciences. For intracellular staining of IFN-γ, TNFα, and IL-2, isolated CD8^+^ T cells were cultured *ex vivo* with a cell Stimulation Cocktail (500X) of phorbol 12-myristate 13-acetate and ionomycin (ThermoFisher Scientific) in the presence of brefeldin A (Sigma) for 6 h at 37 °C. For intracellular cytokine detection, cells were stained with surface antibodies then fixed in 4% paraformaldehyde (Electron Microscopy Services) and permeabilized before staining with intracellular antibodies. The Foxp3 transcription factor staining kit (BD) was used for intracellular staining of TCF-1 and Ki-67. For Annexin V staining, cells were stained in Annexin V Binding buffer according to the manufacturer’s instructions (BioLegend). All samples were acquired on an Accuri C6, FACSAria II (BD Biosciences) or CytoFLEX (Beckman Coulter), and data analyzed using FlowJo V10 software.

### 2.3 Adoptive cell transfer and viral infection

2 x 10^4^ CD45.1^+^ CD8^+^ T cells (for 4 days post infection (dpi) harvest) or 5 × 10^5^ P14 CD45.1^+^ CD8^+^ T cells for cell harvest at 7, 28, and 40 days after infection were adoptively transferred into congenic wild-type CD45.2^+^ C57BL/6 recipient mice, followed by intraperitoneal infection 1 d later with 2 × 10^5^ plaque-forming units per mouse of LCMV-Armstrong or intravenous infection 1 d later with 1 × 10^6^ plaque-forming units per mouse of LCMV-clone 13. LCMV titers were determined by plaque assays as described previously (28–30).

### 2.4 Virus plaque assays

Vero cells were seeded in 6-well plates at 3 x10^5^ cells per well in Dulbecco’s Modified Eagle’s Medium (DMEM) supplemented with 10% FBS and 1% penicillin-streptomycin and L-glutamine. The next day, the culture medium was removed and 0.5 ml of serially diluted sera harvested from LCMV-Armstrong or LCMV-clone 13 virus infected mice was added to each well. Following 1 hour of incubation at 37 °C, the 0.5 ml of serially diluted sera was removed and cells in each well were overlaid with 2 ml of 1% agarose mixture containing Minimum Essential Medium (MEM) supplemented with 14% FBS and 1% penicillin-streptomycin and L-glutamine and incubated at 37 °C. Six days later, 1 ml of 9.375% formaldehyde in PBS was added to each well for 1 hour at room temperature. After 1 hour, the agarose layer was removed from each well and 2 ml of 1% crystal violet was added to each well for 5 minutes. Each well was washed with distilled water and airdried overnight to count plaques.

### 2.5 Multiplex pro-inflammatory cytokine analysis

The V-PLEX Proinflammatory Panel1 (mouse) Kit (1 plate) (Meso scale Discovery, MSD) was used. Dilutions (1 in 2) of sera derived from 12 male and 12 female recipient mice at 7 dpi with LCMV-Armstrong after sex-matched adoptive CD45.1^+^ CD8^+^ T cell transfers, and 6 male and 5 female recipient mice at 7 dpi with LCMV-clone 13, were loaded in duplicate onto the MSD plate. Data acquisition of the MSD plate was performed according to the manufacturer’s instructions. Analysis of cytokine production was disaggregated by sex using GraphPad Prism software. Unpaired t-test was applied for statistical analysis comparisons between the sexes for each infection type.

### 2.6 Cell sorting

Splenocytes were isolated from naïve P14 CD45.1^+^ or wild-type recipient mice at 4 and 7 days after infection with LCMV-Armstrong or LCMV-clone 13 viruses. CD8^+^ T cells were isolated using the CD8^+^ T Isolation Kit (Miltenyi). P14 CD45.1^+^ CD8^+^ T cells were sorted on a FACSAria II (BD Biosciences) for downstream cDNA library generation.

### 2.7 Single-cell cDNA library generation for scRNA-sequencing

**Figure 1:**
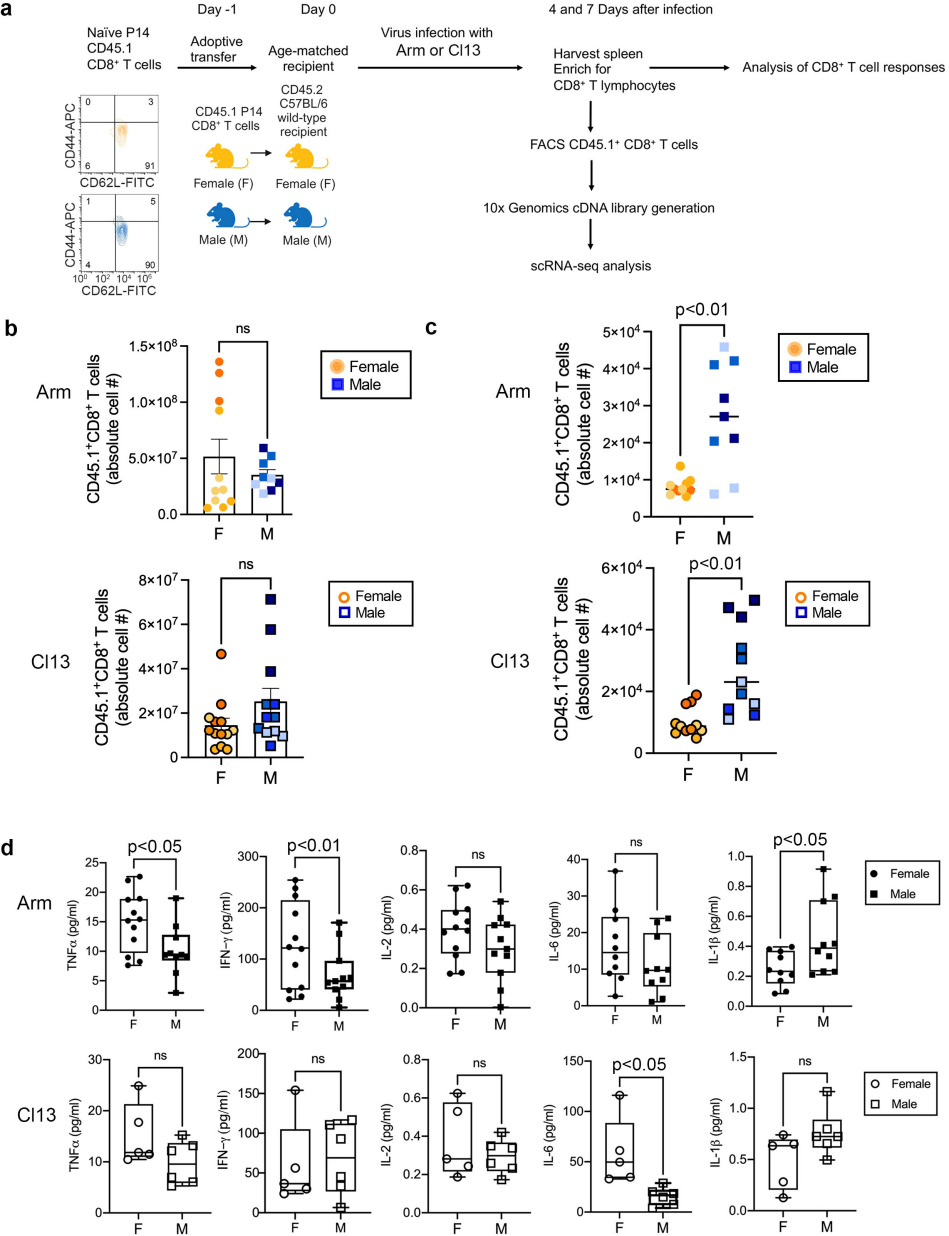
Sex-related CD8^+^ T cell responses to LCMV-Armstrong and LCMV-clone 13 infection. **(a)** Experimental approach (Image created with biorender.com). Flow cytometry staining of CD62L^hi^CD44^lo^ naïve T cells (gated on CD45.1^+^CD8^+^ T cells) is shown. F, female; M, male; Arm, LCMV-Armstrong virus; Cl13, LCMV-clone 13 virus; FACS, fluorescence activated cell sorting; scRNA-seq, single-cell RNA-sequencing. **(b)** Absolute cell numbers of transferred CD45.1^+^ CD8^+^ T cells that are female (F, circles) or male (M, squares), analyzed in the spleen of LCMV-Armstrong (top) or LCMV-clone 13 (bottom) infected mice at 7 dpi. Summary of three independent experiments for LCMV-Armstrong (*n* = 11 mice) and four independent experiments for LCMV-clone 13 (*n* = 12 female mice, *n* = 14 male mice). Independent experiments are represented by different shades of orange (female) and blue (male). * p<0.05, significant, ns, not significant, determined using an unpaired two-tailed Student’s *t*-test. Arm, LCMV-Armstrong virus; Cl13, LCMV-clone 13 virus. **(c)** Absolute cell numbers of transferred CD45.1^+^CD8^+^ T cells that are female (F, circles) or male (M, squares) in the blood at 7 dpi with LCMV-Armstrong (top) and LCMV-clone 13 (bottom) viruses. Independent experiments are represented by different shades of orange (female) and blue (male). Summary of three LCMV-Armstrong and four LCMV-clone 13 independent experiments with at least three mice per group. *** p<0.001, ns, not significant, determined using an unpaired two-tailed Student’s *t*-test. Arm, LCMV-Armstrong virus; Cl13, LCMV-clone 13 virus; dpi, days post-infection. **(d)** MSD, Meso Scale discovery analyses of pro-inflammatory cytokines in the sera of LCMV-Armstrong (top, closed circles, closed squares) or LCMV-clone 13 (bottom, open circles, open squares) infected F, female (circles) or M, male (squares) mice at 7 dpi. Summary of two independent experiments with five to twelve mice per group. Box and whiskers plots show minimum and maximum values and line at the median. Each symbol represents an individual mouse. Error bars indicate standard error of the mean (SEM). ** p<0.01, * p<0.05, ns, not significant, statistical significance of differences was analyzed between female and male samples per infection type, determined using unpaired two-tailed Student’s *t*-test.

6.0 × 10^3^ to 1 × 10^4^ P14 CD8^+^ T cells sorted by FACS were loaded onto the Chromium Next GEM Chip G (10x Genomics) according to the manufacturer’s instructions. P14 CD45.1^+^ CD8^+^ T cells for scRNA-seq were from the same cohort of mice used for phenotypic characterization (**Figure 1a**), *n* = 4 mice per group. Cell lysis, single-cell barcoding and gel-emulsion (GEM) generation were performed in the Chromium Controller (10x Genomics). GEM reverse transcription was performed on the ABI 7500 Thermal Cycler (ThermoFischer Scientific) according to the manufacturer’s instructions. Full-length cDNA from the GEM-RT reaction was purified using silane magnetic beads (10x genomics). Barcoded, full-length cDNA was amplified via PCR on the Veriti 96 Well Thermal Cycler. Illumina Single Cell 3’ complementary DNA (cDNA) libraries were generated of all groups were generated in parallel according to the manufacturer’s instructions (10x Genomics). Quality control measures of the single-cell cDNA libraries were performed on the 2100 Bioanalyzer (Agilent Technologies) and Qubit 4.0 Fluorometer (Thermo Fisher Scientific). Single-cell cDNA libraries were sequenced (50,000 paired-end reads, single-indexing) on an Illumina NovaSeq 6000 at Génome Québec (Montreal, Quebec).

### 2.8 ScRNA-seq data analysis

Sequencing and raw read pre-processing were performed by Génome Québec (Montreal, Quebec). For each library, 237–680 million 100-bp paired end reads were sequenced on an Illumina NovaSeq 6000. Reads were aligned to the GRCm38/mm10 mouse reference genome and quantified using 10X Genomics Cell Ranger. All downstream analyses were performed in R version 4.3.2 (31) using the Seurat v5.0.1 package (32); plots were made with scCustomize v2.0.1 (33). To highlight global variation between samples, UMAPs were projected from the top 10 principal components calculated from the normalized, scaled counts. To account for differences in sequencing depth and library size in each sample, scTransform v0.4.1 was used to further scale gene counts (34). Only cells with greater than 100 reads were analyzed. Cell clusters were identified using the FindNeighbors and FindClusters functions, based on the top 10 PCs, resulting in 12 clusters of cells (resolution=0.2).

### 2.9 Differential expressed genes

Differentially expressed genes (DEGs) were identified using the FindMarkers function with a minimum log_2_ fold change of 0.5, expression in at least 10% of cells of either sample, and an adjusted p-value less than 0.01 (adjusted using Bonferroni correction). Volcano plots of DEGs were generated using the EnhancedVolcano package (35).

### 2.10 Gene ontology analysis

GO term enrichment analysis was performed using topGO (36), using Fisher’s exact test (p < 0.05). Only terms from the “biological processes” ontology were selected. All heatmaps were scaled to provide a z-score (value minus the mean, divided by the standard deviation), hierarchically clustered with “hclust”, and plotted using ggplot2 (37).

### 2.11 Statistical analysis

Statistical analysis was performed using GraphPad Prism software, version 9.0.1. The statistical significance of differences among groups was determined by performing unpaired two-tailed Student’s *t*-test, Mann-Whitney U test, or multi-comparison analysis with one-way ANOVA, Wilcoxon rank sum tests, or Pearson correlation analysis, as indicated in the figure legends, in GraphPad Prism software, version 9.0.1. Error bars represent standard error of the mean (SEM). The p values in the figures indicate the following: *p<0.05, ** p<0.01, *** p<0.001, **** p<0.0001, and ns is not significant (p>0.05).

## 3 Results

### 3.1 Characterization of male and female CD8+ T cell responses to acute versus chronic viral infection

To characterize the cellular responses of male and female CD8^+^ T cells responding to acute-resolving versus chronic viral infection, male or female P14 CD45.1^+^ CD8^+^ T cells were adoptively transferred into age- and sex-matched 6-8-week-old wild-type recipients 1 day before infection with either LCMV-Armstrong or LCMV-clone 13 viruses (**Figure 1a**). Donor P14 CD45.1^+^ female and male mice were progeny from the same dam and sire, and recipient CD45.2 female or male mice housed in same-sex cages. Naïve female and male CD8^+^ T cells showed similar frequencies of CD62L and CD44 protein expression (CD62^hi^CD44^lo^) prior to adoptive cell transfers (**Figure 1a**). Basal frequencies of CD8^+^ (CD8a) and CD4^+^ T cells (CD4), B cells (CD19), and dendritic cells (CD11c) were analyzed by flow cytometry in wild-type naïve C57BL/6 female and male recipient mice (**Supplementary Figure 1**) and exhibited similar trends as previously reported (14, 38, 39). At 7 days post-infection (dpi) with LCMV-Armstrong, we observed a variation in the expansion of both adoptively transferred female and male CD45.1^+^CD8^+^ T cells in the spleen, while an overall increase in expanded adoptively transferred male compared to female CD45.1^+^CD8^+^ T cells was observed after LCMV-clone 13 infection, albeit with a pattern of variation of male CD8^+^ T cell expansion similar to the expanded female and male CD45.1^+^CD8^+^ T cells in LCMV-Armstrong infection (**Figure 1b**; **Supplementary Figure 2**). We found a significant difference in expanded CD45.1^+^CD8^+^ T cells between the sexes in the blood in both infection types (**Figure 1c**), with no difference in the cell contraction kinetics observed between the sexes in the blood in either infection type. We confirmed that virus infection with LCMV-Armstrong and LCMV-clone 13 led to virus clearance and a chronic infection, respectively, as described previously (40). Moreover, short-lived effector cells (SLECs), phenotypically characterized as KLRG1^hi^IL-7R^lo41^and memory precursor cells (MPECs) (41) identified as KLRG1^lo^IL-7R^hi41^ CD45.1^+^CD8^+^ T cells were detected in the spleen at 7 dpi with LCMV-Armstrong (**Supplementary Figures 3a, b**). Female responding CD8^+^ T cells showed slightly higher KLRG1^hi^IL-7R^lo^ cell frequencies compared to responding male CD8^+^ T cells, while a significant increase in KLRG1^lo^IL-7R^hi^ cell frequencies was observed by responding male CD8^+^ T cells (**Supplementary Figures 3a, b**). It should be appreciated, however, that neither KLRG1 and IL-7R expression, while are useful markers, are sufficient for short-lived effector differentiation or memory cell development (42, 43), and remains a caveat of using such cell surface markers to accurately detect effector and memory precursor cells in acute infection. At 40 dpi with LCMV-Armstrong, we found no sex-related difference in the frequency of central (CD62L^hi^CD44^hi^) and effector (CD62L^lo^CD44^hi^) memory cells in the spleen (**Supplementary Figure 3c**). Memory cells in LCMV-Armstrong infection were phenotypically identified as described previously (44, 45). The variation in the frequencies of expanded male compared to female CD8^+^ T cells in LCMV-clone 13 infection and of both sexes in LCMV-Armstrong infection (**Figure 1b**) in the spleen was observed across independent experiments (detailed in **Figure 1b**, symbols are color-coded according to independent experiments for each infection type). It is possible that the variability in the expansion capacities of CD8^+^ T cells by both sexes may be due to influences of different levels of sex steroids (e.g. estrogen, progesterone, androgens) on T cell division, proliferation, and apoptosis (46) in the mice during infection. Sex steroid induced signaling may be tissue-specific as sex steroid levels can differ between different tissues (47), which may explain the differences in the expansion frequencies of female CD8^+^ T cells observed in the spleen versus the blood following LCMV-Armstrong infection.

**Figure 2:**
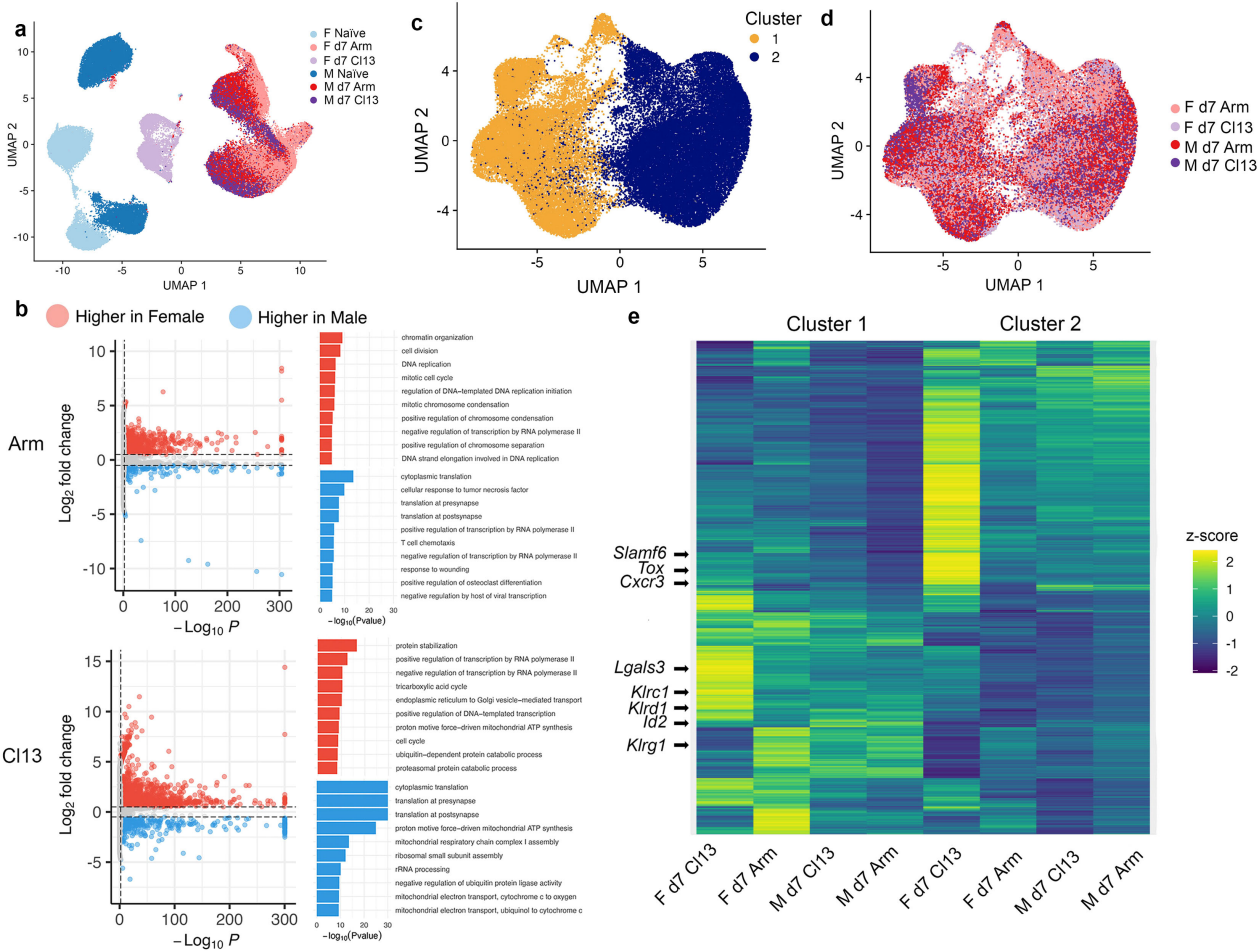
scRNA-seq analysis of male and female CD8^+^ T cells responding to LCMV-Armstrong and LCMV-clone 13 infection. **(a)** UMAP visualization of single CD8^+^ T cells obtained as in **Figure 1**, among naïve female (light blue) 15,462 cells, naïve male (dark blue) 15,114 cells, female day 7 LCMV-Armstrong (pink) 14,933 cells, male day 7 LCMV-Armstrong (red) 15,178 cells, female day 7 LCMV-clone 13 (lavender) 7081 cells, and male day 7 LCMV-clone 13 (purple) 11,796 cells. F, female; M, male; d, day; Arm, LCMV-Armstrong virus; Cl13, LCMV-clone 13 virus. Summary of two independent scRNA-seq experiments with 4 mice per group. **(b)** GO, Gene Ontology analysis of enriched biological processes by differentially expressed genes (absolute average log fold-change > 0.25, adjusted *P*-value < 0.05) between female and male CD8^+^ T cells responding to LCMV-Armstrong (top) and LCMV-clone 13 (bottom) 7 days post-infection. Arm, LCMV-Armstrong virus; Cl13, LCMV-clone 13 virus. **(c)** UMAP analysis of two clusters (cluster 1, yellow, cluster 2, dark blue) of all activated CD8^+^ T cells at 7 dpi with LCMV-Armstrong and LCMV-clone 13 viruses. dpi, days post infection. **(d)** Cell annotation of clusters as in **(c)** of female day 7 LCMV-Armstrong (pink), male day 7 LCMV-Armstrong (red), female day 7 LCMV-clone 13 (lavender), and male day 7 LCMV-clone 13 (purple). **(e)** Heatmap of genes differentially expressed between clusters 1 and 2 in **Figure 2c**.

We next analyzed a panel of pro- and anti-inflammatory cytokines in the sera of LCMV-Armstrong and LCMV-clone 13 infected mice at 7 dpi. In both infection types, increased TNFα, IFNγ, IL-2, and IL-6 was found in the sera of female compared to male mice (**Figure 1d**), while IL-1β was increased in males compared to females (**Figure 2d**). In mice (48) and humans (49), males have exhibited higher production of IL-1β compared to females, suggesting male immune cells may be predisposed to generate stronger IL-1β responses than females. Overall, these findings demonstrate an increase in pro-inflammatory cytokine production by females compared to males during LCMV infection and suggest the possibility that sex-related differences in cell-intrinsic and -extrinsic factors affect CD8^+^ T cell expansion kinetics during LCMV-Armstrong and LCMV-clone 13 infection, representing an area for future experimental study.

### 3.2 Distinct sex-related differences in single-cell CD8^+^ T cell transcriptional profiles in acute versus chronic infection

As we observed sex-related differences in CD8^+^ T cell expansion and pro-inflammatory cytokine expression in both infection types, we next sought to investigate potential sex-related differences in the transcription profiles of CD8^+^ T cells responding to acute and chronic LCMV infection. CD45.1^+^ CD8^+^ T cells were isolated by magnetic bead enrichment and FACS sorted from naïve uninfected P14 (CD62L^hi^CD44^lo^CD45.1^+^CD8^+^), or LCMV-Armstrong and LCMV-clone 13 infected (CD45.1^+^CD8^+^) spleens at 7 dpi (**Figure 1a**). Barcoded single-cell (sc) cDNA libraries were generated (10X Genomics) from inactivated naïve and antigen-specific CD8^+^ T cells harvested at 7 dpi with LCMV-Armstrong or LCMV-clone 13 viruses. All sc-cDNA libraries were generated from the cell populations obtained as in **Figure 1b**, including the same pool of naïve male and female CD45.1^+^ CD8^+^ T cells used for adoptive cell transfers. For each time-point and infection type as in **Figure 1a**, CD45.1^+^CD8^+^ T cells from 4 mice per sex were used and all sc-cDNA libraries were generated in parallel. The sc-cDNA libraries of naïve and activated antigen-specific CD8^+^ T cells were pooled and sequenced at 50,000 paired-end reads on an Illumina NovaSeq6000 (**Figure 1a**; **Supplementary Figure 4**).

To investigate the transcriptional differences between male and female CD8^+^ T cells responding to acute versus chronic infection, we analyzed the scRNA-seq data from antigen-specific naïve female (15,462 cells), naïve male (15,114 cells), activated female (14,933 cells) and male (15,178 cells) CD8^+^ T cells from 7 dpi with LCMV-Armstrong, and activated female (7081 cells) and male (11,796 cells) CD8^+^ T cells from 7 dpi with LCMV-clone 13, and performed Uniform Manifold Approximation and Projection (UMAP) analysis. While all naïve CD8^+^ T cells clustered separately from CD8^+^ T cells isolated from both infection types, female (light blue) and male naïve (dark blue) CD8^+^ T cells formed individual clusters (**Figure 2a**). Naïve female and male CD8^+^ T cells clustered separately based on disparate proportions of mitochondrial derived reads (**Supplementary Figure 5**). In response to LCMV-Armstrong infection, female CD8^+^ T cells (pink) formed a small ridgeline cluster adjacent to the larger cluster of male CD8^+^ T cells (red). Conversely, in response to LCMV-clone 13 infection, female CD8^+^ T cells (lavender) formed a distinct cluster separate from the male CD8^+^ T cell cluster (purple). Interestingly, while male CD8^+^ T cells formed clusters in close proximity in both infection types, female CD8^+^ T cells formed different clusters in response to LCMV-Armstrong infection compared to LCMV-clone 13 infection (**Figure 2a**). The striking differences between female CD8^+^ T cell responses to acute versus chronic infection and between female and male CD8^+^ T cell responses to LCMV-clone 13 infection suggest both cell intrinsic- and extrinsic cues of female CD8^+^ T cells are manifested differently in acute versus chronic infection.

Our data highlights the under-appreciation of sex-related differences in the transcription profiles of naïve CD8^+^ T cells and their transcriptional differences after acute and chronic viral infection. Gene Ontology (GO) analyses of enriched biological pathways in naïve female compared to male CD8^+^ T cells included cytoplasmic translation, rRNA processing, and RNA splicing, whereas enriched biological pathways in naïve male compared to female CD8^+^ T cells included cellular response to type II interferon and protein stabilization (**Supplementary Figure 6**). GO analyses of enriched biological pathways in activated female compared to male CD8^+^ T cells in LCMV-Armstrong infection included chromatin organization, cell division, and DNA replication (**Figure 2b**). In response to LCMV-clone 13 infection, protein stabilization, positive and negative regulation of transcription pathways were enriched in female compared to male CD8^+^ T cells (**Figure 2b**). In both infection types, cytoplasmic translation, translation at presynapse, and translation at post-synapse pathways were enriched in responding male, but not female CD8^+^ T cells (**Figure 2b**), highlighting similarities in male CD8^+^ T cell responses to acute and chronic infection and differences in female CD8^+^ T cell responses to acute versus chronic infection.

UMAP visualization of female and male CD8^+^ T cells responding to LCMV-Armstrong and LCMV-clone 13 at 7 dpi identified two clusters (**Figure 2c**) that included cells from both sexes and infection types (**Figure 2d**), highlighting the heterogeneity in gene expression at 7 dpi overall. We performed UMAP visualization of these cells alone, which partitioned the cells into two clusters (**Figure 2c**). Interestingly, many of the genes that distinguished these clusters were most highly expressed in female CD8^+^ T cells responding to LCMV-clone 13 infection (**Figure 2e**; **Supplementary Figure 7**). In particular, high expression of genes associated with exhaustion, such as *Slamf6 (*50), *Klrc1*, *Klrd1*, *Id2*, and *Tox (*51, 52), and genes which encode for proteins involved in effector CD8^+^ T cell migration and activation, such as *Cxcr3(*CXCR3*) (*53) and *Lgals3* (Galectin-3) (54) were upregulated in the female CD8^+^ T cell clusters derived from LCMV-clone 13 infection (**Figure 2e**), whereas gene expression of the effector associated gene, *Klrg1 (*1) was upregulated in a female CD8^+^ T cell cluster from LCMV-Armstrong infection compared to the other clusters of both infection types (**Figure 2e**). Similarly, at the protein level, KLRG1 expression was significantly higher on responding female CD8^+^ T cells from LCMV-Armstrong infection compared to their male counterparts as well as with responding female and male CD8^+^ T cells from LCMV-clone 13 infection at 7 dpi (**Supplementary Figures 8a-c**). While there were no sex-related differences in the protein expression of Ly108/SLAMF6 by responding CD8^+^ T cells 7 dpi with LCMV-clone 13, we observed a broad distribution of frequencies of SLAMF6 expression of responding female and male CD8^+^ T cells (**Supplementary Figure 8d**), reflecting the heterogeneity in *Slamf6* transcript expression (**Figure 2e**). Our data suggest that different antigen-specific CD8^+^ T cell subsets characteristic of exhausted or effector cellular fates may be specified at an earlier time-point, particularly in female CD8^+^ T cells compared to male CD8^+^ T cells during chronic infection.

### 3.3 Early sex-related transcriptional differences in CD8^+^ T cells responding to chronic LCMV infection

**Figure 3:**
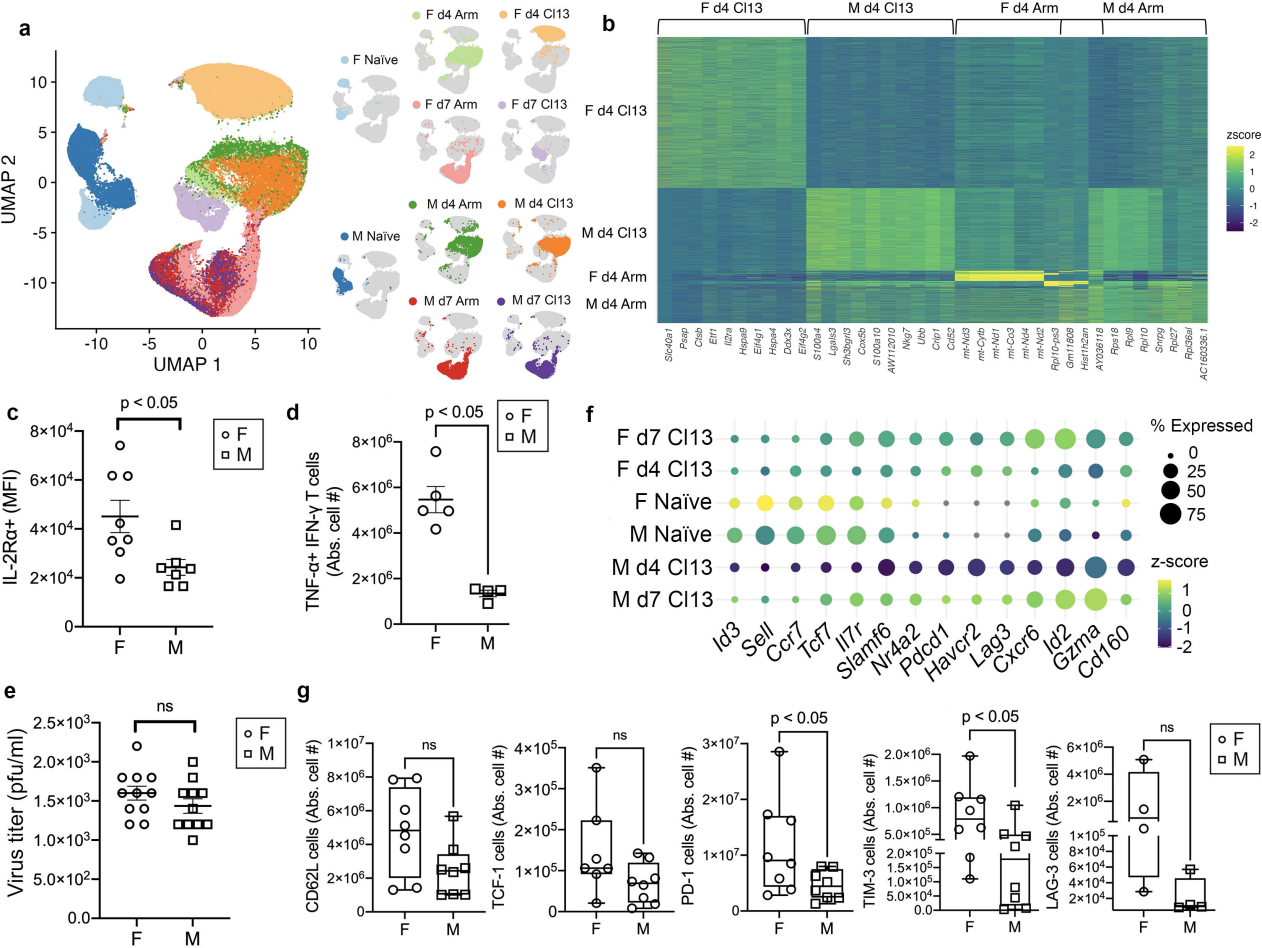
Early transcriptional sex-related differences in CD8^+^ T cells responding to LCMV-clone 13 infection. **(a)** UMAP visualization of single CD8^+^ T cells obtained as in **Figure 1** among naïve female (light blue) 15,462 cells and male (dark blue) 15,114 cells, female day 4 LCMV-Armstrong (light green) 4, 343 cells, male day 4 LCMV-Armstrong (dark green) 7, 398 cells, female day 4 LCMV-clone 13 (light orange) 33, 892 cells, male day 4 LCMV-clone 13 (orange) 18, 531 cells, female day 7 LCMV-Armstrong (pink) 14,933 cells, male day 7 LCMV-Armstrong (red) 15,178 cells, female day 7 LCMV-clone 13 (lavender) 7081 cells, and male day 7 LCMV-clone 13 (purple) 11,796 cells. F, female; M, male; d, day; Arm, LCMV-Armstrong virus; Cl13, LCMV-clone 13 virus. Summary of two independent scRNA-seq experiments with 4 mice per group. **(b)** Heatmap visualization of the top-10 upregulated differentially expressed genes between each population of single CD8^+^ T cells including female day 4 LCMV-clone 13, male day 4 LCMV-clone 13, female day 4 LCMV-Armstrong, and male day 4 LCMV-Armstrong (log FC > 0.5; FDR < 0.01). Three genes were differentially expressed in both female and male day 4 Armstrong, these are noted by overlapping brackets at the top of the heatmap. **(c)** MFI, Mean fluorescence intensity of IL-2Rα protein expression on female and male CD45.1^+^CD8^+^ T cells at 4 days post-infection with LCMV-clone 13, analyzed by flow cytometry. Error bars indicate standard error of the mean (SEM). Summary of two independent experiments, *n* = 8 female mice, *n* = 7 male mice. *p<0.05, determined using an unpaired two-tailed Student’s *t*-test. **(d)** Absolute cell numbers of TNFα^+^IFN-γ^+^ expressing female (open circles) and male (open squares) CD45.1^+^CD8^+^ T cells at 4 days post-infection with LCMV-clone 13, measured by flow cytometry. One of two independent experiments with five mice per group. * p<0.01, determined using an unpaired two-tailed Student’s *t*-test. **(e)** LCMV-clone 13 virus titer in the sera of female (open circles) and male (open squares) mice at 4 days post infection. Summary of two independent experiments with 11 mice per group. pfu, plaque forming units. ns, not significant, determined using an unpaired two-tailed Student’s *t*-test. **(f)** Dot plot of selected T cell exhaustion-associated genes where each dot represents the expression of a gene in the different CD8^+^ T cell population indicated on the horizontal. The size of the dot indicates the proportion of cells in which the transcript was detected and the color indicates the z-score expression of each gene in a population. **(g)** Absolute cell numbers of female (open circles) and male (open squares) CD45.1^+^CD8^+^ T cells expressing CD62L (summary of two independent experiments, *n* = 10 female mice, *n* = 9 male mice), TCF-1 (summary of two independent experiments, *n* = 7 female mice, *n* = 8 male mice), PD-1 (summary of two independent experiments with eight mice per group), TIM-3 (summary of two independent experiments with eight mice per group), and LAG-3 (one of two independent experiments with 4 mice per group) measured by flow cytometry, at 4 days post infection with LCMV-clone 13 virus. Box and whiskers plots show minimum and maximum values and line at the median. *p<0.0001, * p<0.05, ns, not significant, determined using an unpaired two-tailed Student’s *t*-test.

In line with the notion that early programming of CD8^+^ T cell fates may occur during adaptive immunity (12, 44, 55–57), we next investigated whether sex-related differences in the transcription profiles of responding CD8^+^ T cells occurs at an earlier time-point after LCMV-clone 13 infection. To address this, we performed scRNA-seq analysis on female and male antigen-specific CD8^+^ T cells responding to LCMV-clone 13, in comparison to LCMV-Armstrong infection at 4 dpi (**Figure 1a**, **Supplementary Figure 4**). We analyzed the scRNA-seq data from 4 and 7 dpi with LCMV-Armstrong and LCMV-clone 13 together with naïve cells and performed UMAP analyses (**Figure 3a**). Strikingly, female CD8^+^ T cells (light orange) (33, 892 cells) from day 4 LCMV-clone 13 infection formed a single cluster that was distinct from male CD8^+^ T cells (orange) (18, 531 cells) at the same time-point and infection type. In contrast, female (light green) (4, 343 cells) and male (dark green) (7, 398 cells) CD8^+^ T cells from the day 4 LCMV-Armstrong infection formed overlapping clusters (**Figure 3a**) and were in close proximity to the male day 4 LCMV-clone 13 cluster (dark orange), indicating similarities in their transcription profiles.

The female day 4 LCMV-clone 13 cells were characterized by distinct transcriptome profiles relative to all other day 4 samples (**Figure 3b**). Among the top-ranked genes that distinguished female day 4 LCMV-clone 13 cells were *Ddx3x*, which encodes an RNA-binding protein (58), *Ctsb*, encoding cathepsin B, which exerts immunomodulatory functions in microglia (59) and myeloid and programmed cell death pathways (60), and *Il-2ra*, encoding interleukin-2 receptor, alpha subunit (IL-2Rα), a critical component in effector CD8^+^ T cell differentiation, function, and proliferation (61–63) (**Figure 3b**). Mitochondrial encoded genes, including *mtNd3*, *mtCo3*, *mtCytb* were among the top-ranked genes that distinguished female day 4 LCMV-Armstrong cells (**Figure 3b**). With recent studies demonstrating the roles of mitochondrial programming and metabolism in T cell-mediated immunity and exhaustion (64, 65), it remains in question whether sex-specific differences occur in these contexts. As we observed striking sex-related differences in the transcription patterns of d4 LCMV-clone 13 cells, we focused our analysis hereafter on characterizing sex-related differences in CD8^+^ T cells responding to LCMV-clone 13 infection. We confirmed that female CD8^+^ T cells exhibited greater IL-2Rα protein expression compared to male CD8^+^ T cells at 4 dpi with LCMV-clone 13 (**Figure 3c**; **Supplementary Figures 9a, b**). While we did not observe sex-related differences in the frequency of cells expressing the cell proliferation marker, Ki-67, at 4 or 7 dpi with LCMV-clone 13 infection (**Supplementary Figures 10a, b**), we found a significant increase in the frequency of positive Annexin-V (a marker for apoptosis (66)) responding female CD8^+^ T cells compared to male CD8^+^ T cells at 4 dpi, but not at 7 dpi, with LCMV-clone 13 (**Supplementary Figures 10c, d**). This suggests that responding female CD8^+^ T cells undergo increased apoptosis at an earlier time-point after LCMV-clone 13 infection compared to male CD8^+^ T cells, which may explain the increase of expanded male compared to female CD45.1^+^CD8^+^ T cells detected in the spleen and blood at 7 dpi with LCMV-clone 13 (**Figures 1b, c**). Moreover, we found an increase in female responding CD8^+^ T cells expressing pro-inflammatory cytokines, TNFα and IFNγ, compared to male responding CD8^+^ T cells at 4 dpi with LCMV-clone 13 (**Figure 3d**; **Supplementary Figure 9c**). IL-2 can induce TNFα (67) and IFNγ (68) production in activated T cells, which may be augmented by increased IL-2Rα expression and signaling in female compared to male responding CD8^+^ T cells early during LCMV-clone 13 infection. We speculated that differences in LCMV-clone 13 viral titers at early times after infection may, in part, drive sex-related differences in transcription profiles and cytokine production. However, we found no difference in viral titers in the sera of LCMV-clone 13 infected female versus male mice at 4 dpi (**Figure 3e**). Overall, we discovered that female and male CD8^+^ T cells exhibit distinct transcriptional signatures and pro-inflammatory cytokine expression early in the response to a developing chronic infection which may not be driven by early differences in viral antigen stimulation between the sexes.

The intriguing disparate gene expression profiles of early responding female versus male CD8^+^ T cells in LCMV-clone 13 infection, together with our finding that female responding CD8^+^ T cells exhibited high transcript expression of selected exhausted-associated genes at 7 dpi (**Figure 2e**), led us to investigate whether exhaustion-associated genes are expressed differently between the sexes at 4 dpi. Interestingly, we found a striking increase in early expression of genes associated with progenitor exhausted T cells, including *Id3*, *Tcf7* (TCF-1), and *Slamf6*, and terminally exhausted associated inhibitory receptors, *Pdcd1* (PD-1), *Lag-3* (LAG3), and *Havcr2* (TIM-3), in female, but not male CD8^+^ T cells at 4 dpi with LCMV-clone 13 (**Figure 3f**). At 7 dpi with LCMV-clone 13, expression of the selected exhaustion-associated genes appears to be comparable between sexes, suggesting that dynamics in early gene expression regulation may be different between sexes. At the protein level at 4 dpi with LCMV-clone 13, we observed an overall increase in trend of CD62L, TCF-1, PD-1, TIM-3, and LAG-3 expressing female compared to male antigen-specific CD8^+^ T cells (**Figure 3g**; **Supplementarys Figure 11a, b****).** At 7 dpi with LCMV-clone 13, while there were no significant differences in CD62L, TCF-1, PD-1, TIM-3, or LAG-3 expression at the protein level between the sexes (**Supplementary Figure 12**) overall reflecting their transcript expression patterns (**Figure 3f**), we observed a slight increase trend in expression of CD62L, PD-1, and LAG-3 in male compared to female CD45.1^+^CD8^+^ T cells, and the opposite trend for TIM-3 (**Supplementary Figure 12**). Our findings of higher expression of exhaustion associated genes and proteins in female compared to male CD8^+^ T cells at 4 dpi suggest the possibility that female CD8^+^ T cells may adopt a program of exhaustion differentiation earlier than male CD8^+^ T cells during chronic viral infection.

### 3.4 Female CD8^+^ T cells undergo early exhaustion differentiation compared to male CD8^+^ T cells in response to chronic LCMV infection

**Figure 4:**
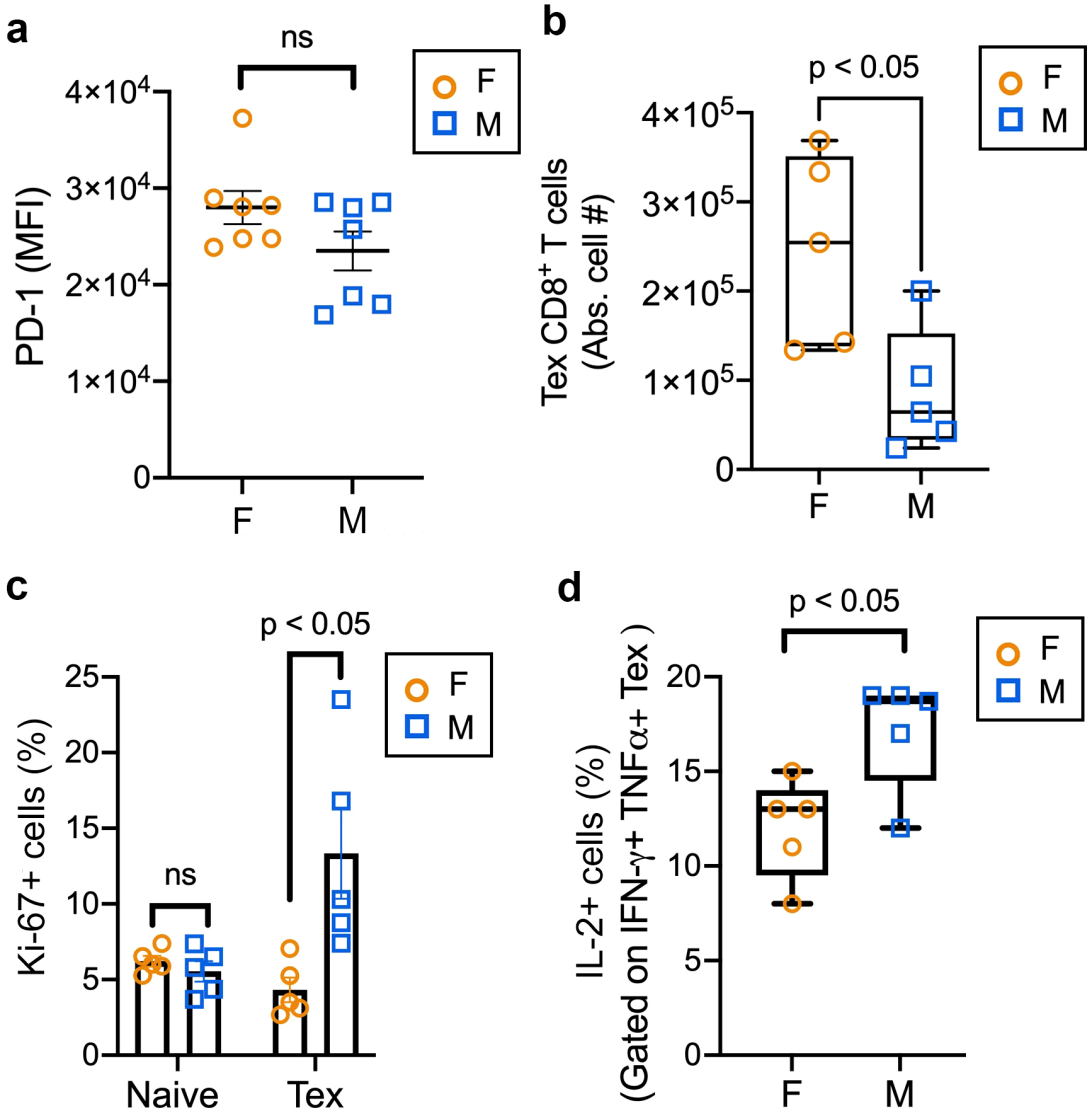
Sex bias in exhausted CD8^+^ T cell differentiation *in vivo*. **(a)** MFI, Mean fluorescence intensity of PD-1 expressing female (orange circles) or male (blue squares) CD45.1^+^CD8^+^ T cells in the spleen at 28 days post infection with LCMV-clone 13, measured by flow cytometry. Summary of two independent experiments with seven mice per group. Error bars indicate SEM, standard error of the mean. ns, not significant, determined using unpaired two-tailed Student’s *t*-test. **(b)** Absolute cell numbers of Tex, terminally exhausted female (orange circles) or male (blue squares) CD45.1^+^CD8^+^ T cells in the spleen at 28 dpi with LCMV-clone 13, measured by flow cytometry. One of two independent experiments with 5 mice per group. Error bars indicate standard error of the mean (SEM). * p<0.05, determined using unpaired two-tailed Student’s *t*-test. **(c)** Frequency (%) of Ki-67^+^ expressing naïve or activated female (orange circles) or male (blue squares) CD45.1^+^CD8^+^ T cells in the spleen at 28 dpi with LCMV-clone 13, measured by flow cytometry. One of two independent experiments with 5 mice per group. Error bars indicate SEM, standard error of the mean . * p<0.05, comparison between female versus male cells at each time-point (naïve, 28 days post infection), determined using an unpaired two-tailed Student’s *t*-test. **(d)** Frequency (%) of IL-2^+^ cells (gated on IFN-γ^+^TNFα^+^ Tex cells) which are female (orange circles) or male (blue squares) Tex, terminally exhausted CD45.1^+^CD8^+^ T cells in the spleen at 28 dpi with LCMV-clone 13, measured by flow cytometry. One of two independent experiments with 5 mice per group. Error bars indicate standard error of the mean (SEM). * p<0.05, determined using unpaired two-tailed Student’s *t*-test.

To examine sex-related differences in exhaustion differentiation, we analyzed exhausted CD8^+^ T cell characteristics of female and male antigen-specific CD8^+^ T cells *ex vivo* 28 days after LCMV-clone 13 infection, a time-point at which exhausted CD8^+^ T cells can be phenotypically identified (8, 50). At 28 dpi with LCMV-clone 13, we observed no difference in PD-1 expression between the sexes (**Figure 4a**; **Supplementary Figure 13**), however found a significant increase in female terminally exhausted (TIM-3^+^PD-1^+^Ly108^lo^) antigen-specific CD8^+^ T cells compared to male CD8^+^ T cells (**Figure 4b**; **Supplementary Figure 14**) and decrease in proliferation of female terminally exhausted CD8^+^ T cells compared to male CD8^+^ T cells (**Figure 4c**). Moreover, we observed a significant decrease in the frequency of IFN-γ^+^TNFα^+^IL-2^+^ expressing female compared to male antigen-specific CD8^+^ T cells, following *ex vivo* stimulation (**Figure 4d**; **Supplementary Figure 14**), suggesting that female CD8^+^ T cells show decreased polyfunctionality than male CD8^+^ T cells at this later time-point. The observation that male CD8^+^ T cells exhibited superior effector capacities at the later time-point after LCMV-clone 13 infection supports our hypothesis that female CD8^+^ T cells undergo an earlier program of exhaustion differentiation compared to male CD8^+^ T cells in this system. Altogether, our data reveals an early sex disparity in exhaustion differentiation kinetics during chronic viral infection.

We next compared the gene signatures of T cell exhaustion profiles from human tumor infiltrating lymphocytes (TILs) previously identified in Zheng et al. (69) with our scRNA-seq data of differentially expressed genes between female and male cells day 7 after LCMV-clone 13 infection. We identified 22 genes in common with positive correlation (Pearson correlation coefficient (*R^2^*) = 0.4) (**Supplementary Figure 15**). Interestingly, all 22 exhaustion-associated genes were upregulated in female compared to male CD8^+^ T cells in our dataset. Of note, 20 of the 22 genes are interferon (IFN) regulated genes (70). These included type I interferon (IFN) regulated genes, *Pdcd1*, *Ifi35*, *Stat3*, *Il2rb, Cxcr6, Park7*; genes regulated by type I and type II (IFN-γ) IFNs, such as *Lag3*, *Tox*, *Cd27*, *Tpi1*, *Pkm*, *Bst2*, *Lyst*, *Acp5*, *Fut8*, *Rab27a*; type II IFN regulated genes, *Prkar1a, Ndfip2*, *Nab1 (*70), and *Vapa*, which encodes for vesicle-membrane-protein-associated protein A (VAPA), previously shown to interact with IFN-inducible antiviral effector protein, IFITM3 (71). In summary, we found that the expression of type I and type II IFN regulated exhaustion genes from human TILs profiles is upregulated in female but not male CD8^+^ T cells at an earlier time-point (7 dpi) after LCMV-clone 13 infection, further demonstrating an early sex-bias in exhaustion-associated transcriptional signatures.

## 4 Discussion

CD8^+^ T cell functional heterogeneity, which can arise early during adaptive immunity (12, 44, 55, 63, 72), mediates protection against infection and chronic stimulation. Here, we sought to gain new insights into previously unknown biological sex-related differences in early CD8^+^ T cell responses and their molecular programs by performing scRNA-seq analysis on male and female CD8^+^ T cells responding to acute and chronic viral infection *in vivo*. Our analyses revealed striking transcriptional differences between male and female CD8^+^ T cells early after chronic but not acute viral infection. Unexpectedly, expression of genes encoding inhibitor receptors and transcription factors associated with the differentiation of exhausted cells was increased in responding female, but not male CD8^+^ T cells at an early time-point (4 dpi) after chronic infection. Moreover, distinct expression of exhaustion and effector associated genes was observed in female responding CD8^+^ T cells at 7 dpi, altogether, suggesting that female CD8^+^ T cells may undergo an earlier program of exhaustion development compared to male CD8^+^ T cells during chronic viral infection. In support of this, we detected an increase of terminally exhausted female CD8^+^ T cells compared to male CD8^+^ T cells and found that responding female CD8^+^ T cells exhibit decreased capacities of proliferation and effector cytokine production compared to their male counterparts at a later time-point after chronic infection.

In this study, we demonstrate the power of sex-disaggregated data analysis to identify sex-related differences in CD8^+^ T cell responses using the LCMV model. While the variability in the expansion of responding female and male CD8^+^ T cells in acute infection and of male CD8^+^ T cells during chronic infection may be in part due to independent experimental effects, our data overall warrants further investigation into CD8^+^ T cell-extrinsic sex differences in the microenvironment, such as sex-specific effects of sex steroids and/or other cytokines, e.g. IL-7 or IL-15, on CD8^+^ T cell responses and differentiation in acute and chronic viral infection. In this study, we did not assess the sex steroid hormone levels of the mice at the times of infection nor at 7 dpi with LCMV-Armstrong or LCMV-clone 13 viruses, however, sex steroid levels and the estrus stage of female mice will be monitored in future studies, as differences in the estrus cycles of female mice, in particular, can affect their susceptibility to infections (73) as well as tissue-specific distribution of sex steroids (47). Following *in vitro* activation of human antigen-specific T cells, estrogen treatment enhances IFN-γ production by male than female CD8^+^ T cells and increases the polyfunctionality of female CD8^+^ T cells in melanoma (74). In non-reproductive tumor models, androgen signaling enhances CD8^+^ T cell exhaustion and its ablation promotes effector activity (23, 24). In contrast, androgen treatment enhances IFN-γ production in CD4^+^ T cells (75). Taken together, the effect of sex steroids on the differentiation and functions of T lymphocytes may be context specific. This, in combination with analyzing the influence of estrogen and androgen on the expression and activities of sex-specific genome encoded genes in T cells, together with delineating sex-steroid induced functions of cells in the tissue niches surrounding T cells (18, 19), highlights the complexity of comprehensively defining sex differences in establishing CD8^+^ T cell diversity.

The striking difference in the transcriptional programs between responding male and female CD8^+^ T cells early during chronic infection suggests there are sex differences in early T cell signaling events. The sex-differential transcriptional programs observed in chronic, but not acute infection may be, in part driven by the infection type. While no difference in viral titers between the sexes was observed in the sera at an early time-point (day 4) after LCMV-clone 13, it is possible that tissue-specific differences in viral loads between female and males may occur, which remains to be investigated. Moreover, possible sex-differential sensitivities to cytokines induced by LCMV-clone 13 infection may impact early transcriptional differences which remains to be studied. LCMV-clone 13 infection leads to higher circulating levels of type I IFNs 24 hrs. post-infection and higher frequencies of infected plasmacytoid dendritic cells (pDCs) compared to LCMV-Armstrong (76, 77). LCMV-clone 13 infection can impair pDC function (77, 78), thus, it is possible that sex-disparities occur in chronic viral infection of pDCs and their cytokine (e.g. IFNs) (79) production, antigen presentation to cognate TCRs, T cell priming, and downstream TCR signaling events. Recently, OT-I transgenic T cells primed *in vitro* with CD11b+ DCs from LCMV-clone 13 infected mice were shown to exhibit repressed effector cytokine production (30); whether there are sex-related differences in this phenomenon remains in question. The increased expression of IL-2Rα by female compared to male CD8^+^ T cells responding to LCMV-clone 13 infection at 4 dpi and increased pro-inflammatory cytokine production by females than males may also contribute to potential sex-related differences in TCR signaling which remain to be explored, as it is known that inflammatory cytokine signaling in CD8^+^ T cells and cell-extrinsic pro-inflammatory signals can directly enhance TCR signaling (80). While we did not observe sex-related differences in animal weight changes during the course of LCMV-clone 13 infection (from d7 to d28) or in the total CD8^+^ T cell frequencies at a later time-point (d28) after LCMV-clone 13 infection (**Supplementary Figure 16**), it remains to be investigated whether there are biological sex differences in the persistence of CD8^+^ T cells and/or other immune cells at time-points later than d28 after chronic LCMV infection, such as the loss of CD4 T cell help (81), which can impact T cell exhaustion development.

Our finding that female CD8^+^ T cells adopt an earlier transcriptional program of exhaustion compared to male CD8^+^ T cells during the course of LCMV chronic infection raises the possibility that female and male T cells are poised differently to generate diverse cellular fates in a controlled manner. Our observation of an exhaustion molecular program is an alignment with recent studies (13, 82), and raises the question whether there are sex differences in epigenetic scarring in T cells (83), which remains to be elucidated. We uncovered previously unappreciated differences in the transcription profiles of naïve female and male antigen-specific CD8^+^ T cells. It is plausible to speculate that resting female and male CD8^+^ T cells are pre-conditioned differently due to X-chromosome encoded genes and/or sex steroid signaling and other factor(s) to be determined, which may predispose a sex-bias in exhaustion development in different tissues or disease contexts. In support of this, Mani and colleagues demonstrated TGF-β pre-conditions naïve T cells in the lymph nodes to enable tissue-resident memory T cell differentiation in the skin (84). Moreover, it remains to be defined whether sex disparities exist in the redistribution of antigen-specific T cells in non-lymphoid organs during chronic viral persistence (85), and organ-specific T cell exhaustion transcriptional signatures (51, 86).

Several immunoregulatory genes are encoded on the X-chromosome (87), positioning females at greater risk for developing autoimmune diseases. A potent capacity of female CD8^+^ T cells to establish exhaustion may be a beneficial tolerance mechanism developed in females to control exacerbated immune responses in autoimmunity and during pregnancy. As such, greater T cell exhaustion is associated with a better prognosis of autoimmune diseases (88–90) and the establishment of effector memory CD8^+^ T cells with hallmarks of exhaustion are critical for a successful pregnancy (91, 92). Further elucidating the molecular mechanisms of sex-divergent candidates we identified in regulating T cell exhaustion formation in autoimmunity and maternal-fetal health may help to uncover common drivers of exhaustion unique to females.

We further found that female responding CD8^+^ T cells have higher expression of exhaustion genes in common with human TILs, compared to their male counterparts at the same time-point (day 7) during a developing chronic infection. Intriguingly, the majority of the common exhausted genes we detected have been shown in studies performed on various cell types to be potentially regulated by type I, type II, or both type I and type II IFNs (70). Type I IFN treatment can regulate expression of co-inhibitory receptors, PD-1, TIM-3, and LAG-3 on human CD4^+^ and CD8^+^ T cells *in vitro (*93), and inhibition of type I IFN signaling at later times after LCMV-clone 13 infection enhances control of the persistent infection (94). Females show enhanced IFN responses and decreased susceptibility to infection than males in general (95), however display greater immune-mediated pathology during chronic infections (96). While a role for type I IFNs in T cell exhaustion has been described previously (12, 81, 93, 94, 97), further studies are needed to better understand the impact of early IFN signaling on CD8^+^ T exhaustion development in a sex-specific manner, and how early type I and type II IFN responses may be induced differentially between females and males during early phases of chronic infection. In support of this, early elevated IFN-γ responses to hepatitis C virus (HCV) infection was associated with early onset of exhaustion by human exhausted CD8^+^ T cells during early phases of infection (98); whether IFN-mediated development of early exhaustion is sex-dependent remains to be elucidated.

Our study revealed sex-related differences in the kinetics of CD8^+^ T cell exhaustion transcription profiles and their functional differences during chronic viral infection. This knowledge is relevant for vaccine development and understanding sex-specific differences in the context of immunotherapy for chronic viral infections and cancers, in particular, immune checkpoint inhibitors which aim to augment CD8^+^ T cell mediated effector anti-tumor functions and re-invigorating T cell exhaustion (99). Sex disparities in the response outcomes to immune checkpoint inhibition have been observed across multiple clinical studies and cancer types (100) with worse adverse effects and survival outcomes in females (101). Fundamentally understanding sex differences in the timing of CD8^+^ T cell exhaustion development, and the cellular mechanisms underlying sex dimorphisms in the immune responses to immune checkpoint inhibition is invaluable for tailoring therapies in a sex-specific manner.

In summary, our findings shed new light on sex differences in early CD8^+^ T cell responses to chronic infection, exhaustion, and T cell biology. This study provides a resource for future work investigating sex-specific mechanisms underlying T cell exhaustion formation. Future studies will aim to elucidate sex differences in early T cell events and IFN-induced molecular mechanisms specifying CD8^+^ T cell exhaustion differentiation, as well as CD8^+^ T cell exhaustion kinetics in other contexts of chronic antigen stimulation, which is significant for the development of sex-specific T-cell based antiviral or immunomodulatory therapies.

## 5 Limitations of the study

This study provided valuable insights into sex-related differences in CD8^+^ T cell responses and their transcriptional programs in a mouse model of acute and chronic viral infection. Our findings derived from a mouse model may not completely reproduce human T cell responses, however, provide basis for future studies using human samples. Another limitation is that sex steroid hormone levels of male and female mice at the time of and during infections, as well as the estrus cycles of the female mice were not assessed, which may impact the host’s infection susceptibility. Future studies should assess these limitations as well as examine tissue-specific distribution of T cell responses to more comprehensively capture sex-related differences in immune responses to infection.

## Data Availability

The datasets presented in this study can be found in online repositories. The names of the repository/repositories and accession number(s) can be found below: https://www.ncbi.nlm.nih.gov/geo/, GSE250251.
