# Supplementary material for: Early sex-related transcriptional differences in CD8+ T cells responding to chronic viral infection reveal a sex bias in exhaustion
Source: Front Immunol. 2026 May 12;17:1783098. doi: 10.3389/fimmu.2026.1783098 (PMC13201411; doi:10.3389/fimmu.2026.1783098)
Supplement: Supplementary file 1 [file DataSheet1.pdf]

Supplementary Figures

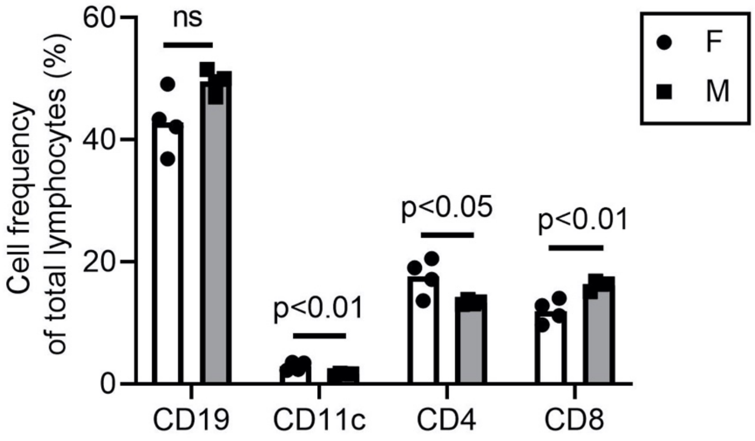

Supplementary Figure 1. Basal frequencies of B cells (CD19), dendritic cells (CD11c), CD4 T cells (CD4) and CD8 T cells (CD8a) in the spleens of naïve uninfected female (closed circles) and male (closed squares) recipient C57BL/6 mice (*n*=4), measured by flow cytometry. One of two independent experiments. ** $p<0.01$, * $p<0.05$, ns, not significant, determined using unpaired two-tailed Student's *t*-test.

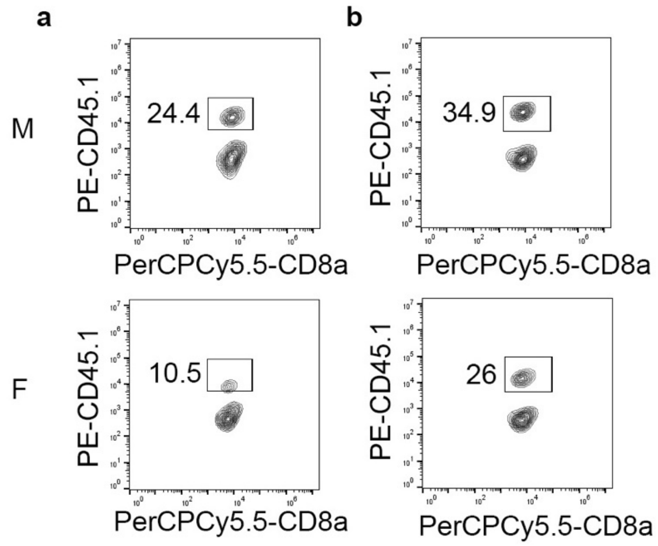

Supplementary Figure 2. Contour plots showing expansion of adoptively transferred male (top) and female (bottom) CD45.1^{+} CD8^{+} T cells in the spleen 7 days after infection with **(a)** LCMV-Armstrong and **(b)** LCMV-clone 13 viruses, relating to Fig. 1b. Shown are cell frequencies (%) analyzed by flow cytometry. M, male; F, female.

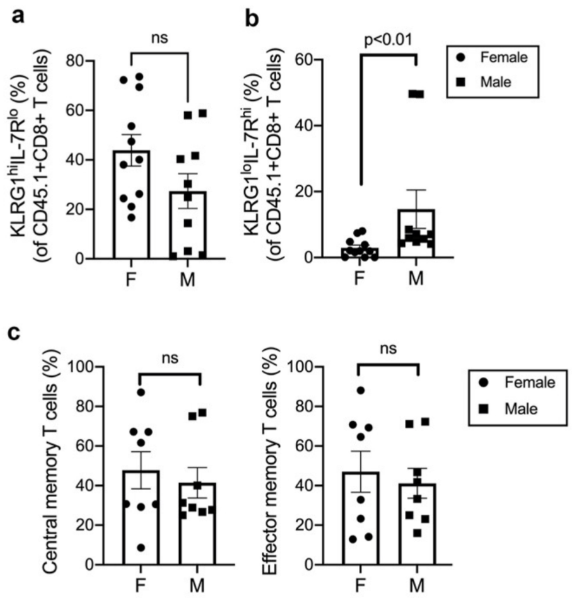

Supplementary Figure 3. Effector and memory differentiation of female and male $CD8^+$ T cells after LCMV-Armstrong infection. Frequency (%) of **(a)** $KLRG1^{hi}IL\text{-}7R^{lo}$, short-lived effector cells (SLEC), and **(b)** $KLRG1^{lo}IL\text{-}7R^{hi}$, memory precursor effector cells (MPEC), gated on female (closed circles) and male (closed squares) CD45.1+ $CD8^+$ T cells at 7 dpi with LCMV-Armstrong in the spleen. **(c)** Memory cells, subdivided into central ($CD44^{hi}CD62L^{hi}$) (left) and effector ($CD44^{hi}CD62L^{lo}$) (right) memory female (closed circles) and male (closed squares) $CD45.1^+CD8^+$ T cells in the spleen at 40 days after infection with LCMV-Armstrong. Summary of two independent experiments with four mice per group. ** $p < 0.01$, ns, not significant, determined by Mann-Whitney U test. Error bars show standard error of the mean (SEM). f, female; m, male; dpi, days post infection.

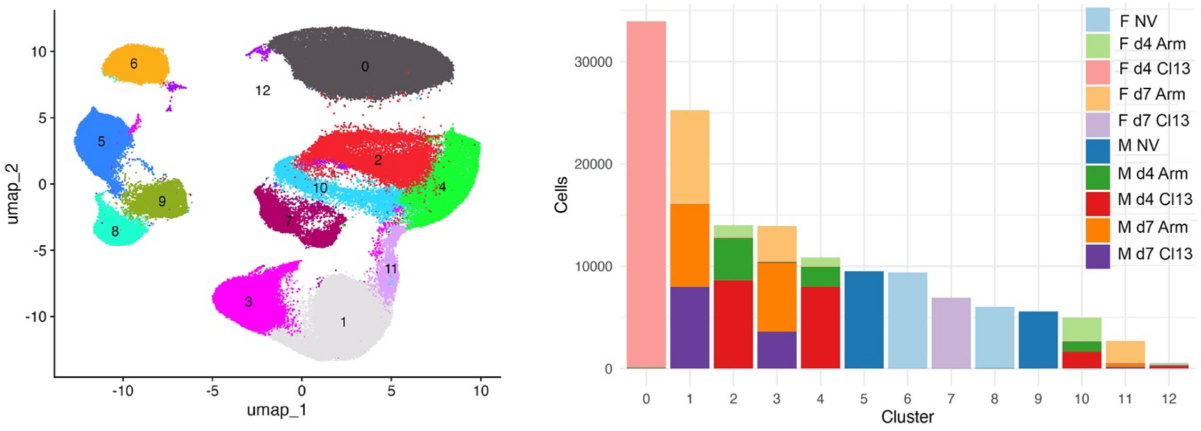

Supplementary Figure 4. Origin of CD8$^+$ T cells per cluster as indicated retained for downstream scRNA-seq analysis after quality control and filtering. UMAP visualization (left) of single male and female naïve and activated CD45.1+ CD8$^+$ T cells from the spleen at days 4 and 7 after LCMV-Armstrong and LCMV-clone 13 infection, detailed in Fig. 2a, Fig. 3a. Stacked bar chart (right) shows the number of cells from each population that were assigned to each cluster of the UMAP (left). F NV, female naïve; M NV, male naïve; Arm, LCMV-Armstrong virus; Cl13, LCMV-clone 13 virus; d4, 4 days post infection; d7, 7 days post infection.

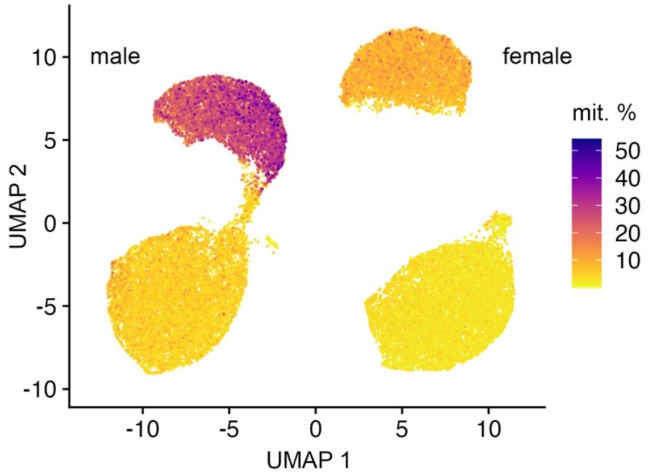

Supplementary Figure 5. Proportion of mitochondrial derived reads in naïve female (right) and male (left) CD45.1+ CD8$^+$ T cells. The colour of each cell corresponds to the proportion of the total number of reads that are mitochondrial derived.

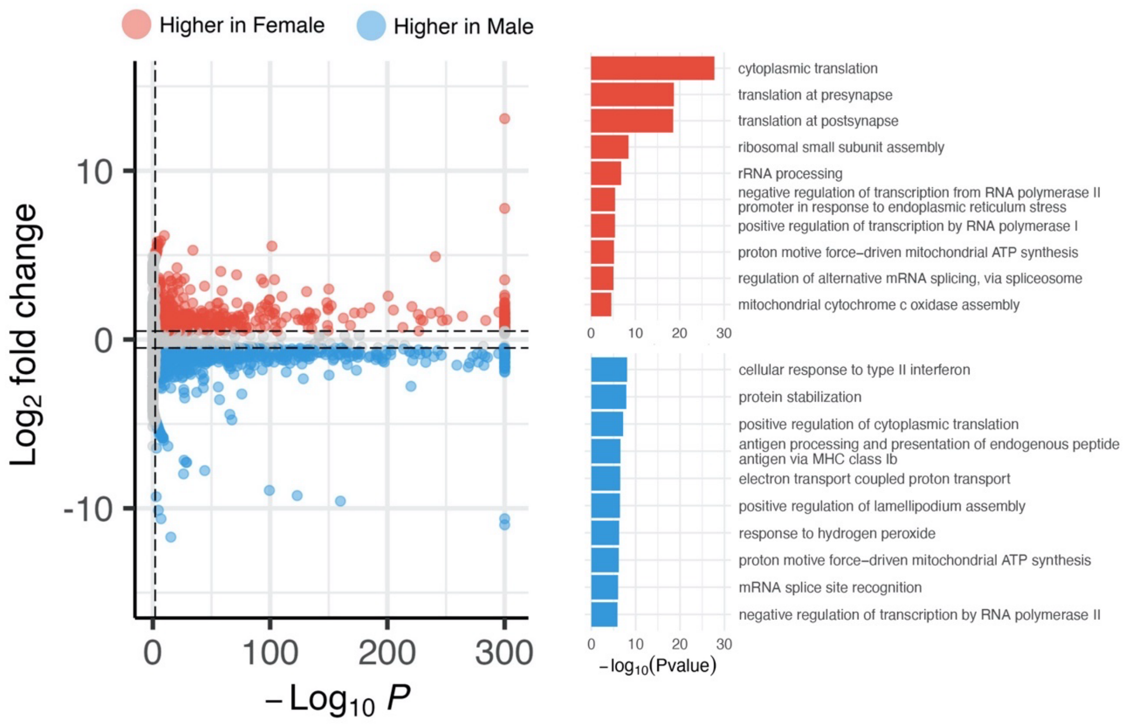

Supplementary Figure 6. Gene Ontology (GO) analysis of enriched biological processes by differentially expressed genes (absolute average log fold-change > 0.25, adjusted *P*-value < 0.05) between naïve female (red) and male (blue) $CD8^+$ T cells.

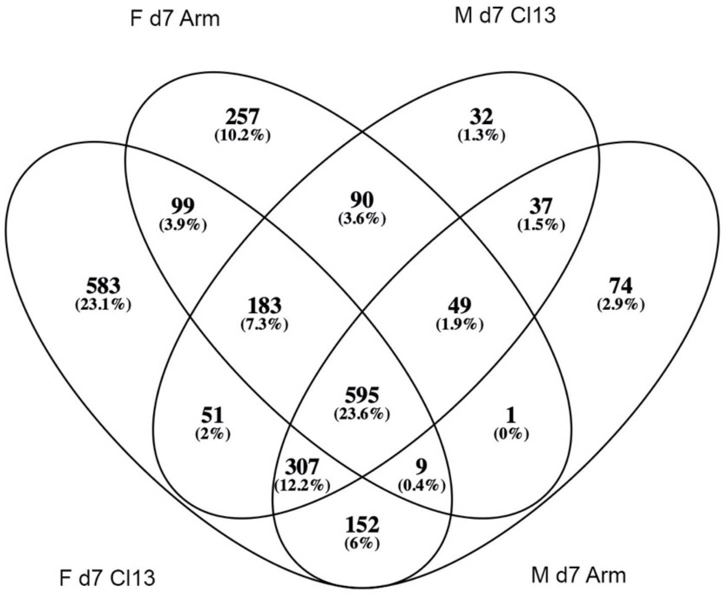

Supplementary Figure 7. Venn diagram of differentially expressed genes between the two clusters of the day 7 single $CD8^+$ T cells, detailed in Fig. 2e. Genes that were up and down regulated are included in this analysis. F, female; M, male; Arm, LCMV-Armstrong virus; Cl13, LCMV-clone 13 virus; d7, days post infection.

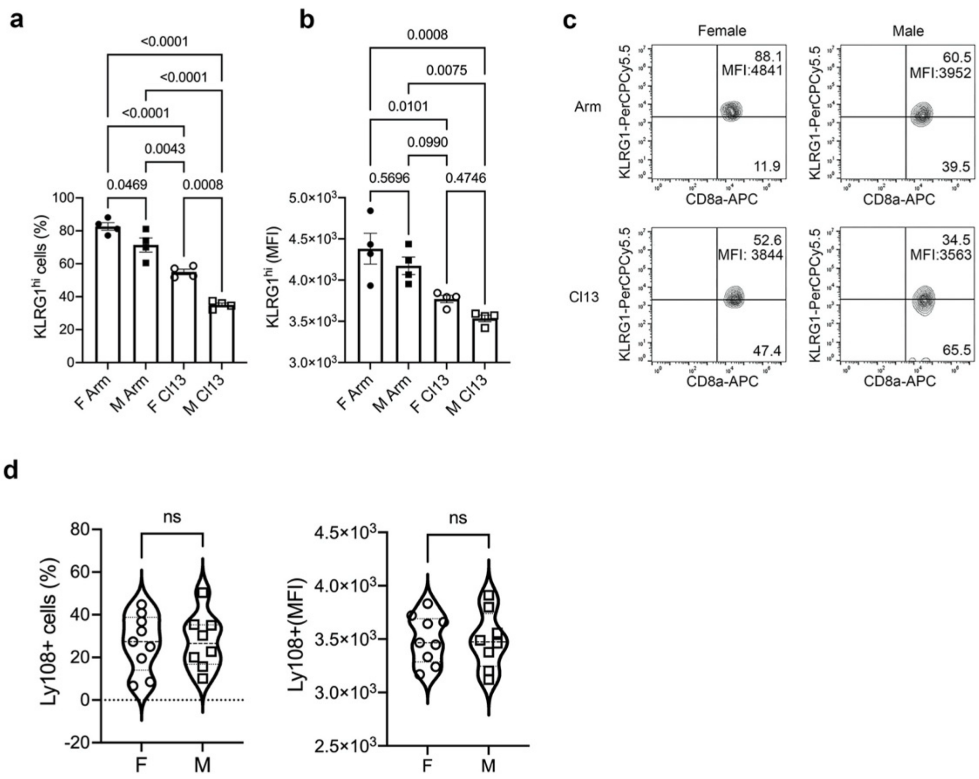

Supplementary Figure 8. (a) Cell frequency (%), **(b)** MFI, and **(c)** contour plots of KLRG1^{hi} expression, gated on female (closed black circles) and male (close black squares) CD45.1+CD8^{+} T cells 7 days after LCMV-Armstrong infection, and on female (open circles) and male (open squares) CD45.1+ CD8+ T cells 7 days after LCMV-clone 13 infection in the spleen, analyzed by flow cytometry. One of two independent experiments with four mice per group. P values are shown, determined using One-way ANOVA test. * p<0.05, ** p<0.01, *** p<0.001, **** p<0.0001, ns, not significant p>0.05. **(d)** Violin plots showing cell frequency (%) (left) and MFI (right) of Ly108+ expression, gated on female (open circles) and male (open squares) CD45.1+ CD8+ T cells 7 days after LCMV-clone 13 infection in the spleen, analyzed by flow cytometry. Summary of two independent experiments with at four to five mice per group. Ns, not significant, determined using unpaired two-tailed Student's *t*-test.

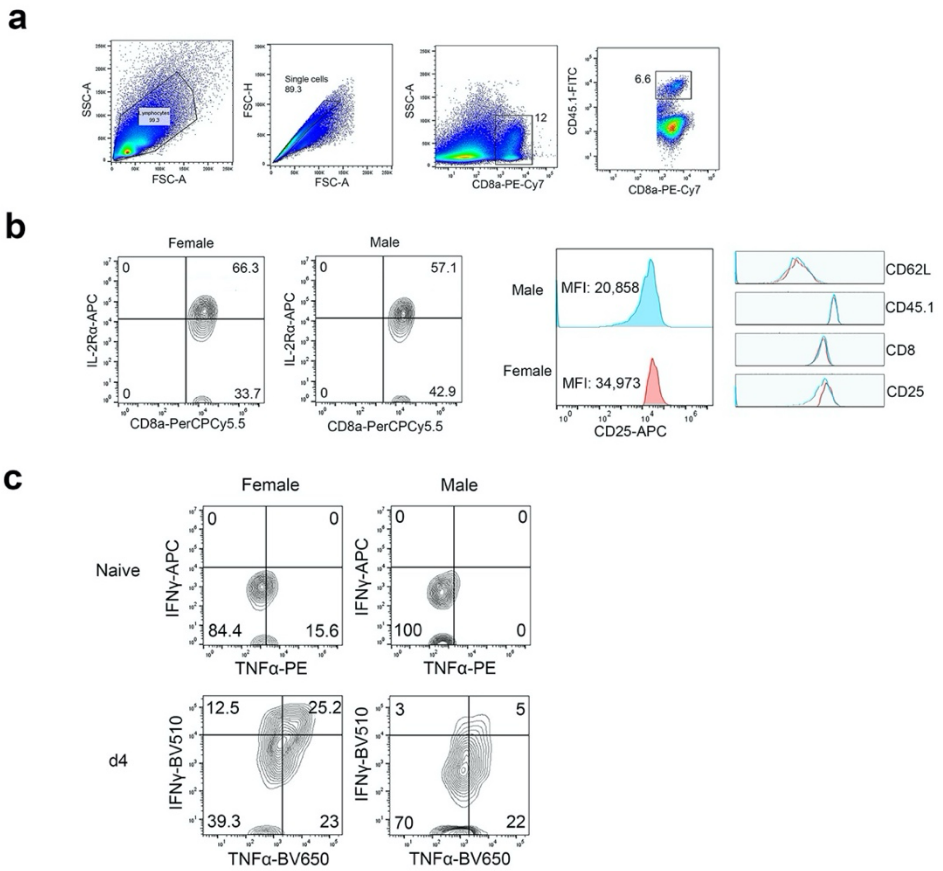

Supplementary Figure 9. Flow cytometry analysis of **(a)** representative gating strategy for flow cytometry analysis of responding CD45.1^{+}CD8^{+} T cells in the spleen 4 days after LCMV-clone 13 infection. Representative **(b)** contour plots (left) showing IL-2Rα/CD25+ cell frequencies (%) and histograms (right) showing MFI of IL-2Rα/CD25 expression on CD45.1+CD8^{+} T cells, gated as in **(a)**, detailed in Fig. 3c. **(c)** Representative contour plots of IFNγ^{+}TNFα^{+} -expressing naïve CD45.1+CD8^{+} T cells (top) and CD45.1+CD8^{+} T cells (bottom) at 4 days after LCMV-clone 13 infection, analyzed by flow cytometry, relating to Fig. 3d. MFI, mean fluorescence intensity; d4, 4 days after LCMV-clone 13 infection.

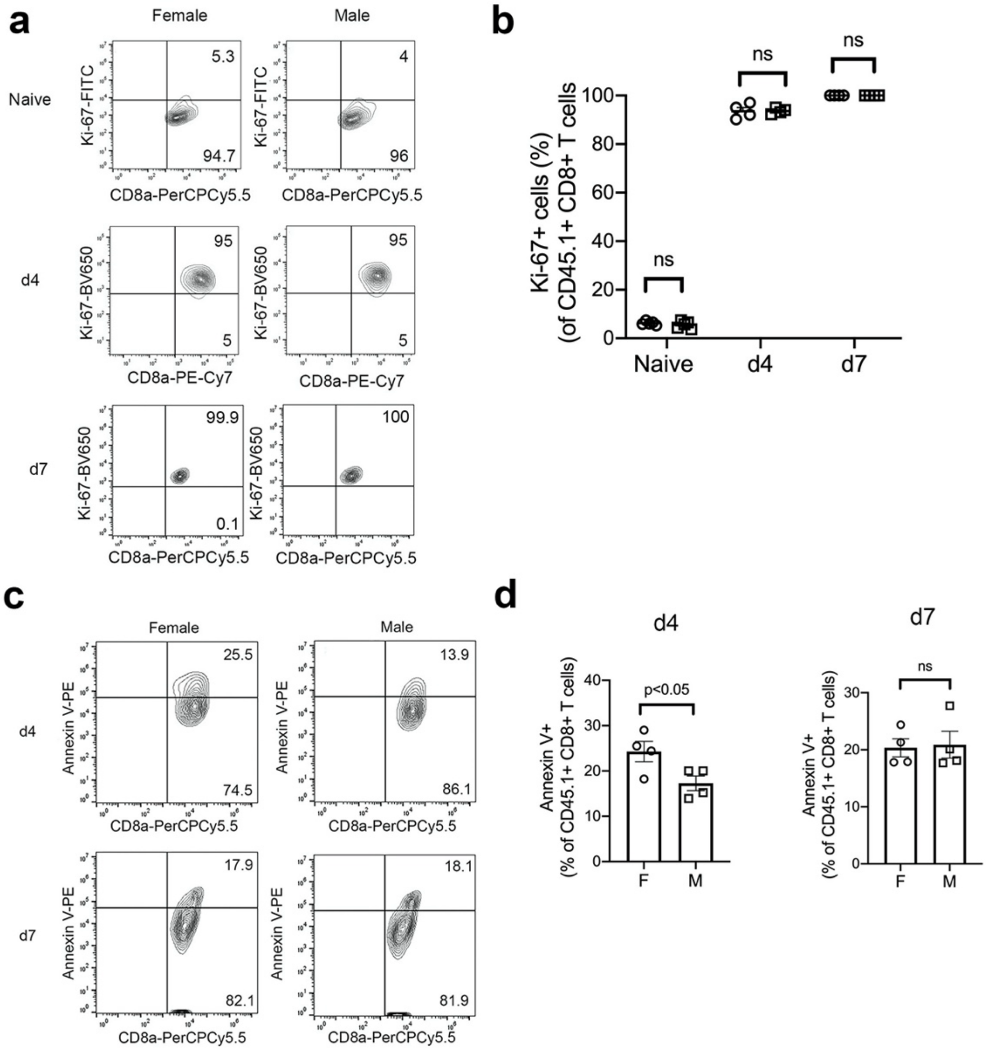

Supplementary Figure 10. (a) Representative flow cytometry contour plots showing frequencies (%) of Ki-67-expressing female (left) and male (right) CD45.1+CD8$^+$ T cells in the spleen that are naïve (top) or analyzed at 4 (middle) and 7 (bottom) days after LCMV-clone 13 infection. **(b)** Cell frequency (%) of Ki-67+ expressing naïve or responding CD45.1+ CD8$^+$T cells at 4 and 7 days after LCMV-clone 13 infection in the spleen, measured by flow cytometry. One of two independent experiments with four mice per group. Ns, non-significant, comparison is between female versus male cells at each time-point (naïve, 4, 7 days post infection), determined using an unpaired two-tailed Student's *t*-test. Error bars indicate standard error of the mean (SEM). d, days

post infection. **(c)** Representative flow cytometry contour plots showing frequencies (%) of Annexin-V+-expressing female (left) and male (right) CD45.1+$CD8^+$ T cells in the spleen at 4 and 7 days after LCMV-clone 13 infection. **(d)** Cell frequency (%) of Annexin-V+ expression by responding CD45.1+ $CD8^+$T cells at 4 (left) and 7 (right) days after LCMV-clone 13 infection in the spleen, measured by flow cytometry. One of two independent experiments with four mice per group. *p<0.05, ns, non-significant, determined using an unpaired two-tailed Student's *t*-test. Error bars indicate standard error of the mean (SEM). d, days post infection.

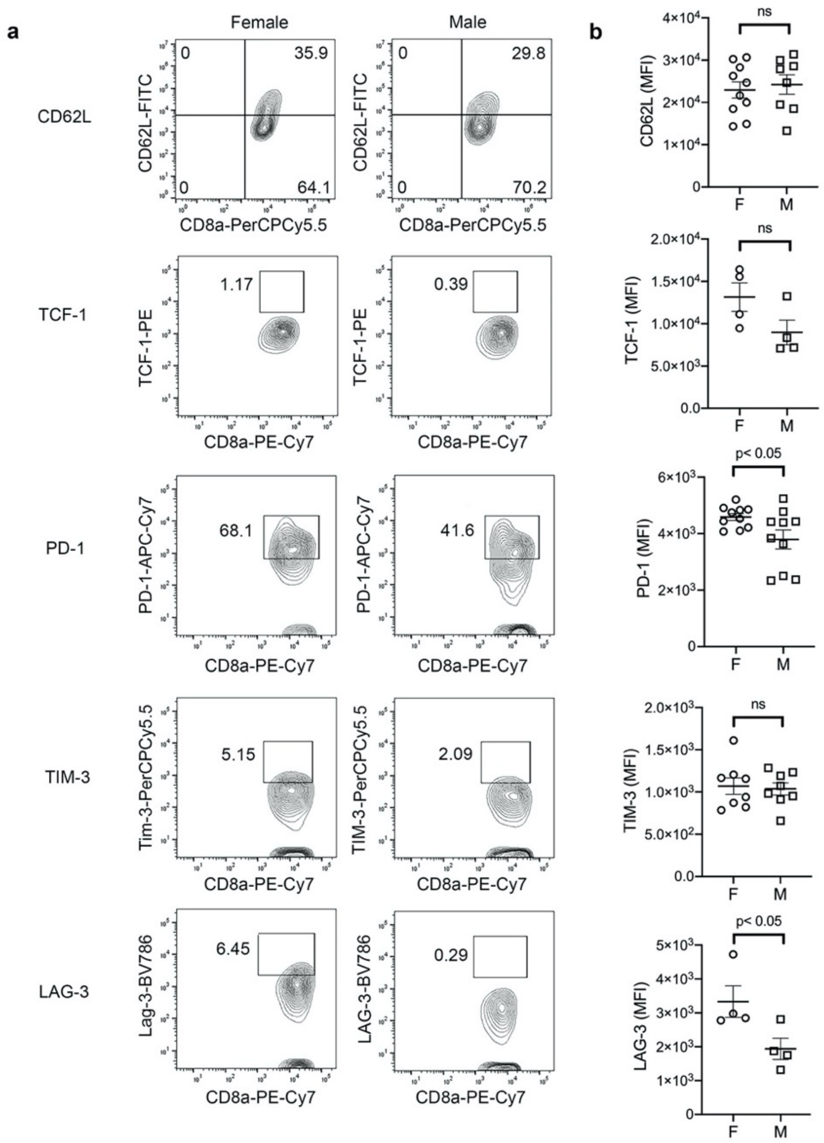

Supplementary Figure 11. Representative flow cytometry **(a)** contour plots showing cell frequency (%) of female (left) and male (right), and **(b)** MFI of CD62L, TCF-1, PD-1, TIM-3, and LAG-3 (from top to bottom) protein expression by female (left, open circles) and male (right, open squares) CD45.1+CD8$^+$ T cells in the spleen 4 days after LCMV-clone 13 infection, relating to Fig. 3g. * p<0.05, ns, not-significant, determined using Student's unpaired t-test. Error bars indicate standard error of the mean (SEM).

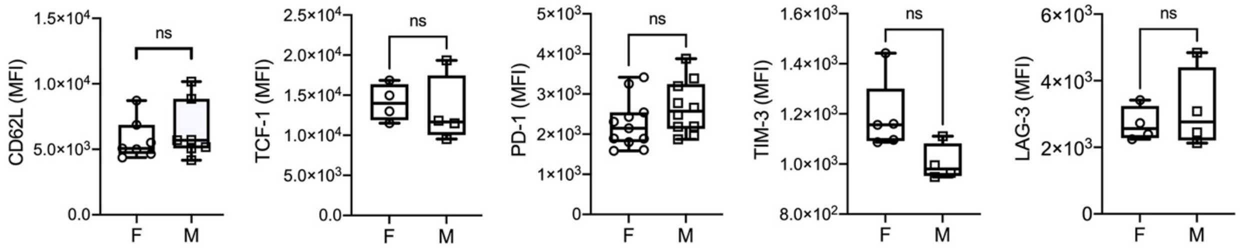

Supplementary Figure 12. Flow cytometry analysis of MFI expression of CD62L, TCF-1, PD-1, TIM-3, and LAG-3 by female (left, open circles) and male (right, open squares) CD45.1+$CD8^+$ T cells in the spleen 7 days after LCMV-clone 13 infection. Box and whiskers plots show minimum and maximum values and line at the median. Box and whiskers plots of CD62L and PD-1 are the summary of two independent experiments with four mice per group. Box and whiskers plots of TCF-1, TIM-3, and LAG-3 are one of two independent experiments with four to five mice per group. MFI, mean fluorescence intensity; F, female; M, male.

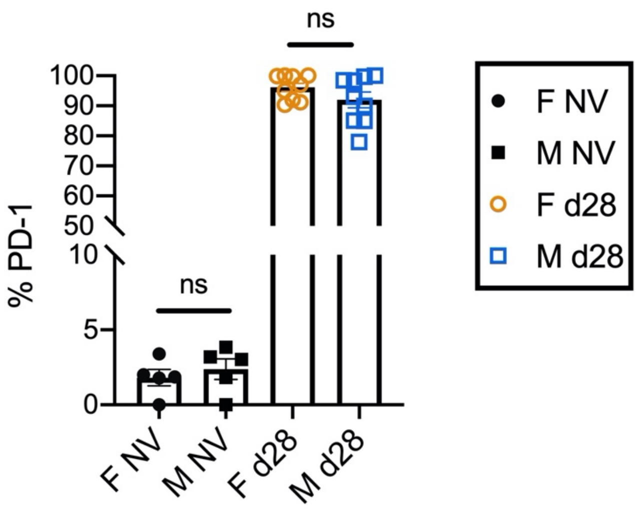

Supplementary Figure 13. Frequency (%) of PD-1 expressing naïve female (closed black circles) or male (closed black squares) and responding female (open orange circles) or male (open blue squares) CD45.1$^+$CD8$^+$ T cells in the spleen 28 days after infection with LCMV-clone 13 virus, relating to Fig. 4a. Data shown for % PD-1 of naïve cells is one of two independent experiments with four mice per group. Data shown for % PD-1 of CD8$^+$ T cells at d28 is the summary of two independent experiments with four mice per group. F, female; M, male; NV, naïve; d, day. Ns, not-significant, determined using Student's unpaired *t*-test. Error bars indicate standard error of the mean (SEM).

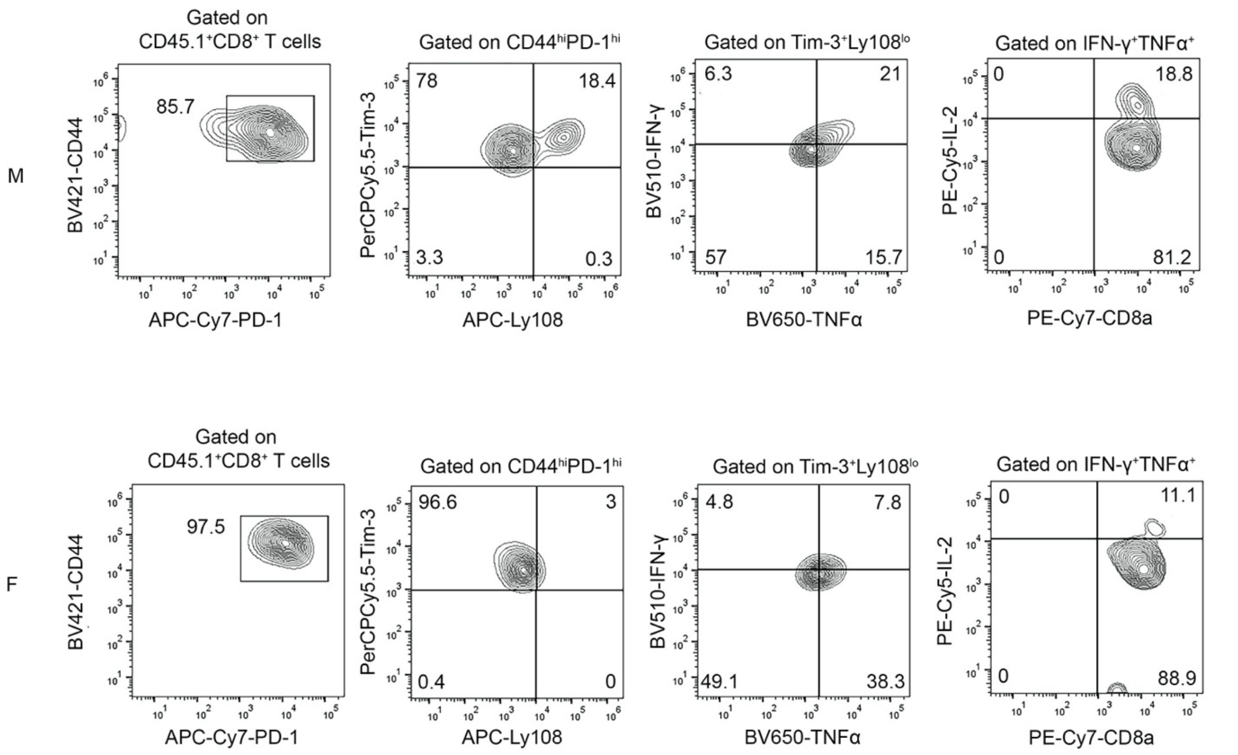

Supplementary Figure 14. Representative flow cytometry gating strategy showing frequencies (%) of terminally exhausted ($CD44^{hi}PD\text{-}1^{+}TIM\text{-}3^{+}Ly108^{lo}$) male (top) and female (bottom) $CD45.1^{+}$ $CD8^{+}$ T cells of and their polyfunctionality ($IFN\gamma^{+}TNF\alpha^{+}IL\text{-}2^{+}$) in the spleen 28 days after LCMV-clone 13 infection, relating to Fig. 4c, Fig, 4d. M, male; F, female.

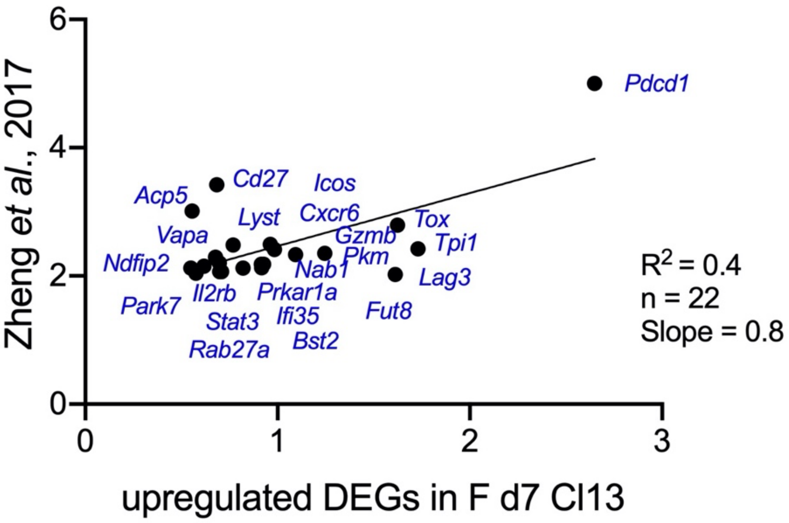

Supplementary Figure 15. Common exhaustion associated genes identified between scRNA-seq derived gene signatures of tumour infiltrating lymphocytes (TILs) previously reported in Zheng et al., 2017 and our scRNA-seq data of differentially expressed genes (DEGs) between female and male CD8$^+$ T cells 7 days after LCMV-clone 13 infection. Each point represents the expression changes of the indicated gene (average log2FC in female versus male scRNA-seq DEGs d7 Cl13, logFC in Zheng et al., 2017). Pearson correlation coefficient (R2), number genes (*n*=22), and slope of the regression line are shown. Linear regression line is shown in black. ** p <0.01, two-tailed, determined by Pearson correlation analysis. F, female; d7, 7 days; Cl13, LCMV-clone 13 virus.

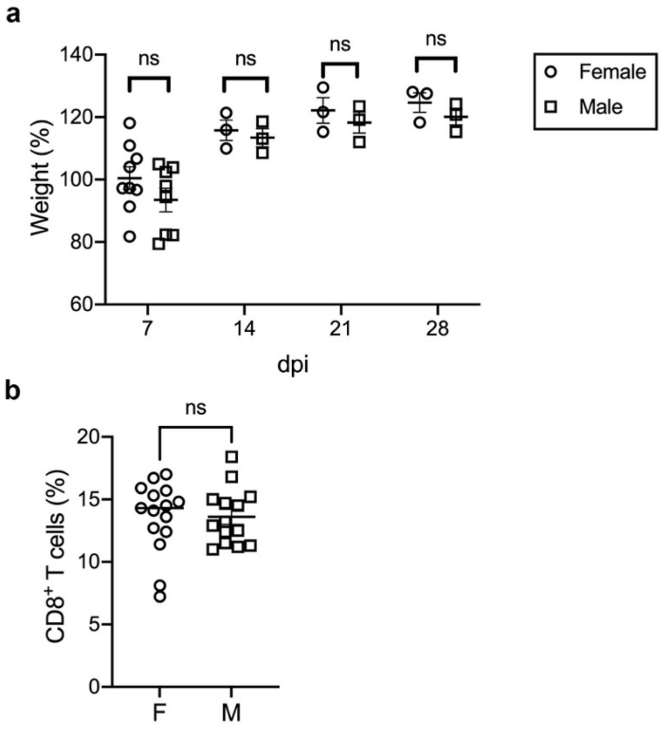

Supplementary Figure 16. Analysis of changes in weight and total CD8$^+$ T cell frequencies (%) in recipient C57BL/6 mice which received CD45.1+CD8$^+$ T cells 1 day prior to infection with LCMV-clone 13. **(a)** Weight change in female (open circles) and male (open squares) C57BL/6 mice at 7, 14, 21, and 28 days after infection with LCMV-clone 13. The percentage weight change for female (open circles) and male (open squares) mice infected with LCMV-clone 13 relative to baseline weight (uninfected at day 0) is shown. Wilcoxon rank sum tests were performed to calculate p-values for each sex grouping at 7, 14, 21, and 28 dpi. Ns, not-significant. One of two independent experiments with three to nine mice per group. Error bars indicate standard error of the mean (SEM). **(b)** Frequency of total female (open circles) and male (open squares) CD8$^+$ T cells in the spleen at 28 dpi with LCMV-clone 13. Summary of three independent experiments with five mice per group. Ns, not significant, determined by Mann-Whitney U test. dpi, days post infection; F, female; M, male.
